# Transmembrane BAX inhibitor motif containing 1 inhibition of lysosomal degradation of TGF-β receptor 1 suppresses cellular senescence and hepatocarcinogenesis

**DOI:** 10.1016/j.jbc.2025.110904

**Published:** 2025-11-04

**Authors:** Daoyu Zhou, Wei Yu, Yating Zheng, Xiaojuan Hou, Kuizhi Zhang, Xiaofeng Qian, Lixia Duan, Shiyao Feng, Mengmeng Xue, Xinyu Zhu, Hengyan Zhang, Luyao Zhang, Lixin Wei, Wenting Liu, Jinghua Jiang, Li Zhang

**Affiliations:** 1 Clinical Research Unit, The First Affiliated Hospital of Naval Medical University, Shanghai, China; 2 Tumor Immunology and Metabolism Center, National Center for Liver Cancer, Naval Medical University, Shanghai, China; 3 Department of Hepatic Surgery, Third Affiliated Hospital of Naval Medical University, Shanghai, China; 4 Department of Clinical Pharmacology, Second Affiliated Hospital of Anhui Medical University, Hefei, China; 5 Department of Pharmacy, Shanghai Putuo District Liqun Hospital, Shanghai, China; 6 Department of Intensive Care Medicine, Shanghai Sixth People's Hospital, Shanghai, China; 7 Department of Urology, Chaohu Hospital of Anhui Medical University, Hefei, China; 8 Department of Medical Oncology, Fudan University Shanghai Cancer Center, Shanghai, China

**Keywords:** cellular senescence, hepatocellular carcinoma, lysosomal degradation, TGF-β signaling pathway, transmembrane BAX inhibition motif containing1

## Abstract

Growing evidence indicates that the cellular senescence (CS) plays a crucial role in hepatocarcinogenesis. Transmembrane BCL-2-associated X protein inhibitor motif containing 1 (TMBIM1) has been shown to inhibit CS. However, the role and mechanism of TMBIM1 associated CS in hepatocarcinogenesis remains unclear. In our study, TMBIM1 was highly expressed in the adjacent tissues of patients with liver cancer and its expression of TMBIM1 gradually decreased during hepatocarcinogenesis in a rat primary liver cancer model. In a *Tmbim1*-overexpressed rat model, the occurrence of hepatocellular carcinoma (HCC) was highly inhibited and overall survival was prolonged. In addition, the aggregation of senescent cells promotes the activation of hepatic progenitor cells to promote the occurrence of HCC. However, the expression of TMBIM1 is negatively correlated with CS. Furthermore, *Tmbim1* downregulated the expression of *Cdkn2a*, *Cdkn1a* and SASP mRNA to inhibit CS. Interestingly, overexpression of *Tmbim1* and knockdown of *Tmbim1* had completely reverse effects on lipopolysaccharide-induced CS. Moreover, under the regulation of TMBIM1, the phosphorylation of Smad2/3 was inhibited, the TGF-β signaling pathway was blocked by the lysosomal degradation of TGF-β type I receptors (TGFBR1) and CS was inhibited. Finally, we demonstrated that the interaction between TMBIM1 and Rab9a contributes to lysosomal degradation of TGFBR1. Together, these data indicate that TMBIM1 interacts with Rab9a to promote degradation of TGFBR1 in lysosomes. In turn, the CS signaling pathway was blocked and the occurrence of HCC was inhibited.

Hepatocellular carcinoma (HCC) is the third leading cause of cancer-related mortality worldwide, with an increasing incidence and high mortality rate (1, 2). Recent data suggest that cellular senescence (CS) is a key factor in the occurrence of the liver cancer (3). During the accumulation of senescent cells, immune cells play a vital role in modulating the tissue microenvironment, contributing to cancer promotion, which is a hallmark of malignant tumors (4, 5). Similarly, senescent hepatocytes are considered markers of chronic liver disease, which is characterized by persistent liver damage, excessive accumulation of scar tissue and cholangiocyte responses (6). Senescent cells can produce various pro-inflammatory and angiogenic factors senescence-associated secretory phenotype (SASP), altering the hepatic tissue microenvironment and increasing the risk of carcinogenesis in adjacent nonsenescent cells (7, 8). Therefore, a deeper understanding of the impact of cellular senescence on liver cancer occurrence has significant scientific and clinical implications for the development of new therapeutic strategies for HCC.

TMBIM1 (transmembrane BAX inhibition motif containing 1) is a conserved sequence protein located on the membranes of late endosomes and lysosomes, with higher expression levels in liver tissue (9). It regulates vascular remodeling by inhibiting the production of matrix metalloproteinase 9 (MMP-9), which is associated with elevated levels of aging and active MMP-9 (10). Research on TMBIM1 in tumors is still in its early stages of development. Previous studies have found a significant correlation between high TMBIM1 expression and the characteristics of serrated polyposis syndrome, which increases the risk of colorectal cancer (11). TMBIM1 is highly associated with colorectal cancer risk in the Chinese population and its expression is significantly higher in colorectal cancer tissues (12). Additionally, TMBIM1 promotes the proliferation of glioblastoma cells and reduces apoptosis by targeting the p38/MAPK pathway (13). Overexpression of *Tmbim1* in the liver can downregulate inflammatory responses through lysosomal degradation targeting TLR4, effectively alleviating steatohepatitis and metabolic disorders induced by a high-fat diet (14). In the realm of research concerning the role of TMBIM1 in HCC, one study has showed that TMBIM expression was induced by exogenous antioxidants N-acetylcysteine and glutathione to promote HCC development and aggravate tumor aggression (15). However, the role of TMBIM1expression in hepatocarcinogenesis remains unclear.

Increasing evidence suggests that TGF-β signaling is multifaceted and associated with age-related diseases, including Alzheimer's disease, muscle atrophy and obesity. TGF-β exhibits strong growth inhibitory activity across various cell types and multiple growth regulatory mechanisms are linked to cellular senescence and stem cell aging (16). The regulation of cellular senescence by TGF-β signaling occurs through the activation of transmembrane BAX inhibitor motif containing 1 (TGFBR1) and TGF-β type II receptor (TGFBR2). Once activated, TGFBR1 phosphorylates Smad2/3, which subsequently forms complexes with Smad4 and translocates to the nucleus to regulate the expression of various target genes (17). TGFBR1 is a critical component which acts as a kinase that phosphorylates and activates the downstream Smad2/3 pathway. Lysosomal degradation of TGFBR1 plays an important role in this signaling pathway (18). Therefore, it is important to investigate whether the expression of TMBIM1 on the membranes of late endosomes and lysosomes can block the TGF-β signaling pathway by promoting the lysosomal degradation of TGFBR1 and inhibiting CS.

In our study, we analyzed the expression levels of TMBIM1 in liver cancer patients and DEN-induced rat liver cancer models and found that TMBIM1 was highly expressed in the adjacent tissues of liver cancer patients and the expression of TMBIM1 gradually decreased during hepatocarcinogenesis in a rat primary liver cancer model. Nevertheless, in a *Tmbim1*-overexpressed rat model, the incidence rate of HCC was significantly decreased and overall survival was prolonged. In addition, the aggregation of senescent cells promotes the activation of hepatic progenitor cells (HPCs) to promote the occurrence of HCC, but the expression of TMBIM1 is negatively correlated with senescent cells. Furthermore, *Tmbim1* downregulated the expression of *Cdkn2a*, *Cdkn1a* and SASP mRNA to inhibit CS. Interestingly, overexpression of *Tmbim1* and knockdown of *Tmbim1* had completely reverse effects on lipopolysaccharide (LPS)-induced CS. Moreover, under the regulation of TMBIM1, the phosphorylation of Smad2/3 was inhibited, the TGF-β signaling pathway was blocked by lysosomal degradation of TGFBR1 and CS was inhibited. Finally, we demonstrated that the interaction between TMBIM1 and Rab9a contributes to lysosomal degradation of TGFBR1. Thus, our findings provide a clear understanding of the relationship between TMBIM1, CS and HCC.

## Results

### TMBIM1 expression is associated with hepatocarcinogenesis

**Figure 1:**
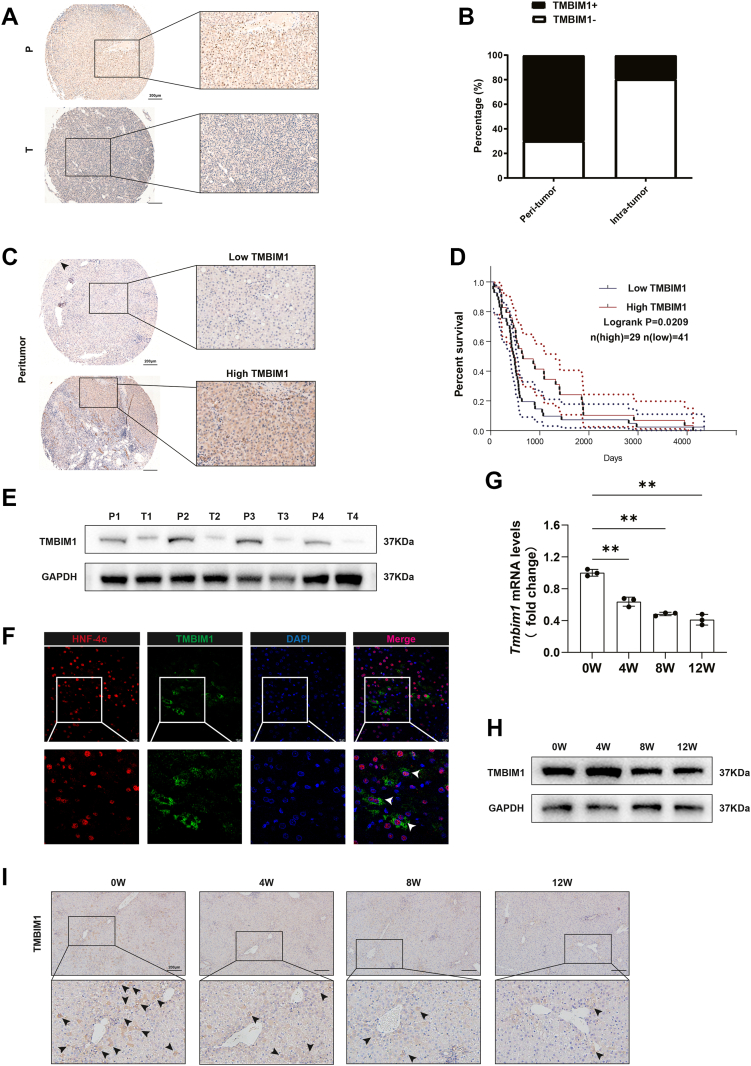
**TMBIM1 expression is elevated in adjacent liver tissues from HCC patients but gradually decreases during hepatocarcinogenesis in a rat model.***A*, immunohistochemical analysis of transmembrane BAX inhibitor motif containing 1 (TMBIM1) expression in a tissue microarray comprising paired peritumoral (P) and hepatocellular carcinoma (T) tissues from patients. *B*, statistical analysis of TMBIM1 immunohistochemical staining intensity in paired adjacent and tumor tissues from HCC patients as shown in (*A*). *C*, classification of adjacent the liver tissues from HCC patients into TMBIM1 high- and low-expression groups based on immunohistochemical results. *D*, Kaplan–Meier analysis of overall survival in patients with high or low TMBIM1 expression in adjacent the liver tissues. *E*, Western blot analysis of TMBIM1 protein levels in paired adjacent and tumor tissues from HCC patients. *F*, immunofluorescence (IF) staining showing colocalization of TMBIM1 and hepatocyte nuclear factor in adjacent liver tissues. *G*, qPCR analysis of Tmbim1 mRNA expression during diethylnitrosamine-induced hepatocarcinogenesis in rats. *H*, Western blot analysis of TMBIM1 protein expression during rat hepatocarcinogenesis. *I*, Immunohistochemical staining of TMBIM1 in the liver tissues at different stages of rat hepatocarcinogenesis. All data are presented as mean ± SD; n = 3. ∗∗*p* < 0.01. TMBIM1, transmembrane BAX inhibitor motif containing 1.

To investigate the expression of TMBIM1 during liver cancer occurrence, we analyzed adjacent nontumor and liver cancer tissues from 70 patients (Table S3). We found that TMBIM1 was expressed at higher levels in the adjacent tissues than in the liver cancer tissues. Immunohistochemical analysis employing liver tissue microarrays demonstrated a positive expression rate of TMBIM1 was approximately 70% in the peritumoral tissues, whereas the rate in liver cancer tissues was only approximately 20% (Fig. 1, *A* and *B*). Furthermore, long-term follow-up of 70 liver cancer patients showed that the overall survival of 29 patients with high TMBIM1 expression in adjacent tissues was greater than that of 41 patients with low TMBIM1 expression (Fig. 1, *C* and *D*). Subsequently, we selected four patients to perform Western blot for the TMBIM1 protein in both adjacent and liver cancer tissues, confirming that the protein expression of TMBIM1 was higher in adjacent tissues (Fig. 1*E*). Given that TMBIM1 is expressed at higher levels in the liver and hepatocytes are the primary cells responsible for liver function, we conducted immunofluorescence(IF) staining on the adjacent tissues and found that TMBIM1-positive cells co-expressed hepatocyte marker hepatocyte nuclear factor (Fig. 1*F*). To further explore the relationship between TMBIM1 and liver cancer occurrence, we established an N-nitrosodiethylamine (DEN)-induced rat liver cancer model with a period of 0 to 12 weeks representing the period of HCC occurrence. The mRNA and protein expression levels of TMBIM1 at the time of 0, 4, 8 and 12 W gradually decreased (Fig. 1, *G* and *H*). Additionally, immunohistochemical staining of liver tissues confirmed that the number of TMBIM1-positive cells decreased as carcinogenesis progressed (Figs. 1*I* and S4*A*). These results suggested that TMBIM1 was associated with HCC occurrence.

### TMBIM1 inhibits hepatocarcinogenesis in rats

**Figure 2:**
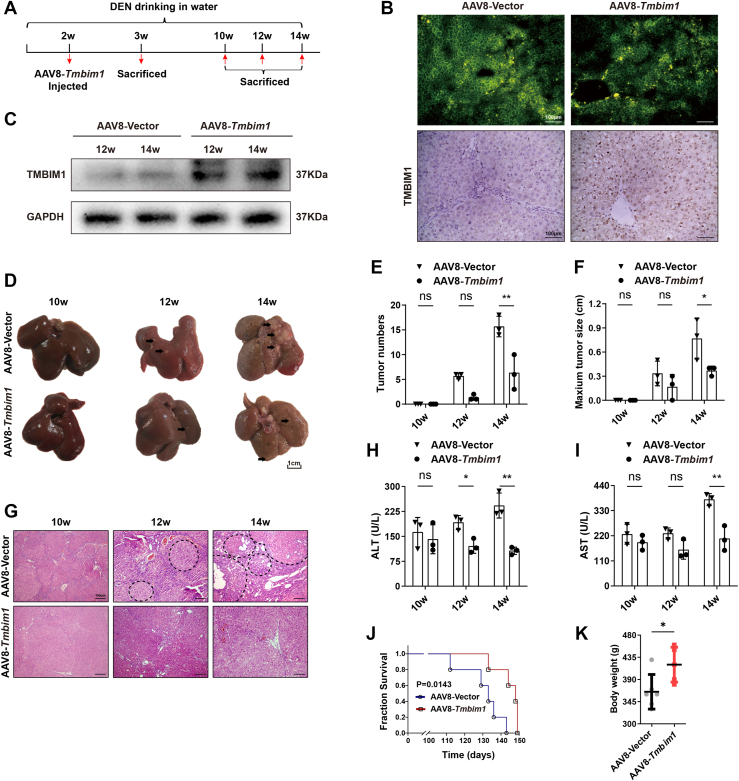
**TMBIM1 suppresses DEN-induced hepatocarcinogenesis in rats.***A*, schematic of the DEN-induced hepatocarcinogenesis model in rats overexpressing Tmbim1. *B*, fluorescence microscopy of frozen liver sections 1 week after tail vein injection of adenovirus, showing viral localization (*upper panel*), and immunohistochemical staining detecting TMBIM1 expression in liver tissue at the same time point (lower panel). *C*, Western blot analysis of TMBIM1 protein expression in the liver tissues at weeks 12 and 14 of DEN-induced hepatocarcinogenesis in Tmbim1-overexpressing rats. *D*, representative macroscopic images of livers at different stages of DEN-induced hepatocarcinogenesis in control and Tmbim1-overexpressing groups. *E*, quantitative analysis of tumor number from the samples shown in (*D*). *F*, analysis of tumor volume during hepatocarcinogenesis. *G*, histopathological changes in the liver during progression of carcinogenesis in rats. *H*, serum ALT levels measured at different stages of carcinogenesis. *I*, serum aspartate aminotransferase levels at indicated time points during DEN-induced hepatocarcinogenesis. *J*, survival analysis of different experimental groups throughout the DEN-induced hepatocarcinogenesis protocol. *K*, statistical analysis of the body weight changes in control and Tmbim1-overexpressing groups during carcinogenesis. All data are presented as mean ± SD; n = 3. ∗*p* < 0.05, ∗∗*p* < 0.01.

Through analysis of human tissue specimens and rat liver cancer models, it was confirmed that the expression of TMBIM1 gradually decreased as HCC occurred and that TMBIM1 is associated with the overall survival of patients with HCC. To investigate the effects of TMBIM1 on HCC, we established a *Tmbim1*-overexpressed rat model by injecting AAV8-*Tmbim1* adenoviruses at the second week after initiation of DEN-induction (Fig. 2*A*). A week after injecting AAV8-*Tmbim1* and AAV8-Vector adenoviruses, the fluorescence images of the frozen sections from liver tissues demonstrated the adenovirus accumulated in the liver (Fig. 2*B*, upper). Additionally, there are more TMBIM1-positive cells in the AAV8-*Tmbim1* group compared to the control group (Fig. 2*B*, lower panel, and Fig. S4*B*). At 12 W and 14 W of carcinogenesis, the TMBIM1 expression in the AAV8-*Tmbim1* group increased significantly (Fig. 2*C*), indicating *Tmbim1* was overexpressed. In this model, TMBIM1 inhibited the occurrence of HCC, as evidenced by better liver condition, fewer tumor nodules and smaller sizes in the AAV8-*Tmbim1* group at 10, 12 and 14 weeks (Fig. 2, *D*–*F*). At the 12-weeks mark of the carcinogenesis study, neoplastic growths were evident in the control cohorts, characterized by disrupted tissue architecture and a plethora of vacuolar degenerations. In stark contrast, hepatocytes in the AAV8-*Tmbim1* groups exhibited a more orderly arrangement, with a conspicuous absence of significant tumorigenesis and the preservation of a distinct, well-preserved hepatic lobular pattern. (Fig. 2*G*). Compared with the control group, the levels of alanine aminotransferase and aspartate aminotransferase significantly reduced in the AAV8-*Tmbim1* group, indicating that TMBIM1 can improve liver function and body weight in rats (Fig. 2, *H*, *I* and *K*). Additionally, it prolonged the survival period of rats (Fig. 2*J*), which is consistent with the finding that TMBIM1 can improve the overall survival of patients with liver cancer.

During HCC occurrence, inflammation, liver fibrosis and cirrhosis occur in conjunction with apoptosis. There was a significant reduction in collagen deposition and the number of α-smooth muscle actin-positive cells in *Tmbim1*-overexpressed rats’ liver tissue (Figs. S1, *A*, *B* and S4*M*). Also, inflammatory factors (interferon-γ (IFN-γ), IL-2, interleukin-6 (IL-6), tumor necrosis factor-α (TNF-α) and interleukin-1β (IL-1β)) and CD68-positive macrophages decreased notably (Figs. S1, *C*, *D* and S4*N*). The number of apoptotic cells marked by caspase 8 and Fas/CD95 decreased (Figs. S1, *E*, *F* and S4, *O*, *P*), which was consistent with terminal deoxynucleotidyl transferase dUTP nick-end labeling staining (Fig. S1*G*). These results suggested that TMBIM1 inhibited liver cancer progression.

### TMBIM1 is negatively associated with CS and CS plays a role in the occurrence of liver cancer

**Figure 3:**
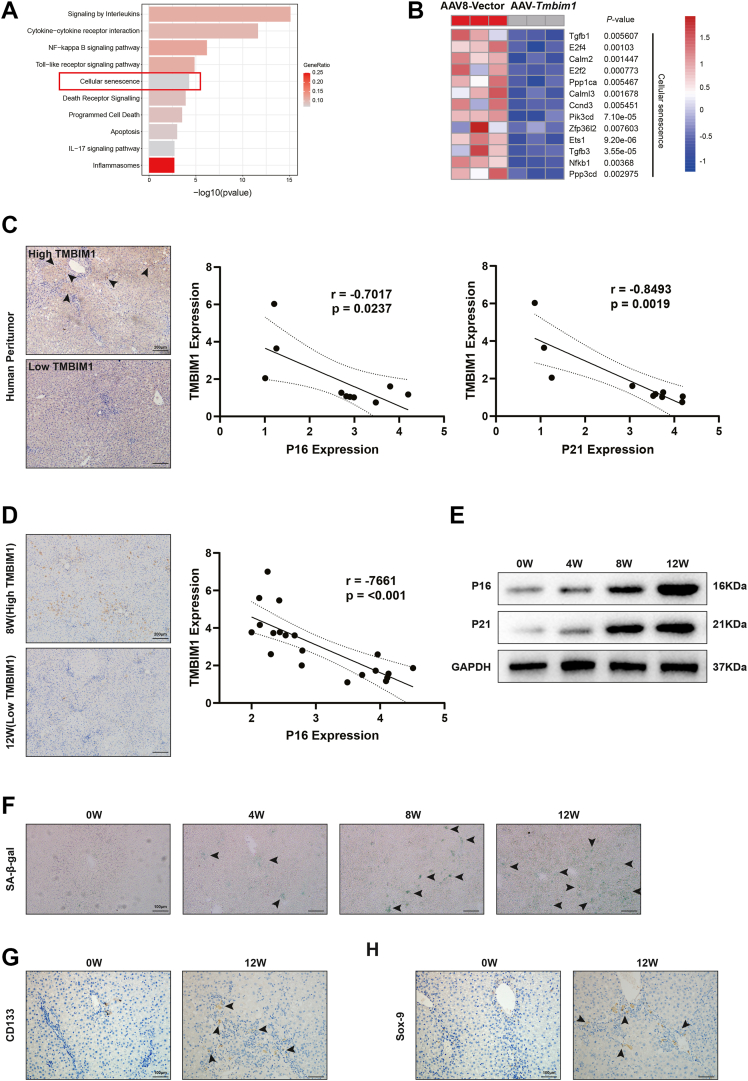
**TMBIM1 is negatively correlated with cellular senescence.***A*, KEGG pathway enrichment analysis of differentially expressed genes in liver tissues from rats overexpressing TMBIM1 at 12 weeks after carcinogen induction. *B*, heatmap displaying senescence-associated genes among the differentially expressed genes. *C*, immunohistochemical analysis of TMBIM1, cyclin-dependent kinase inhibitor 2A (P16), and cyclin-dependent kinase inhibitor 1A (P21) expression in hepatocellular carcinoma (HCC) tissues from patients; correlation analysis was performed to evaluate the relationship between TMBIM1 and the senescence markers P16 and P21. *D*, immunohistochemical detection of TMBIM1 and P16 expression in liver tissues of rats at 0 weeks and 12 weeks after carcinogen induction; correlation analysis was conducted based on expression levels. *E*, Western blot analysis of P16 and P21 expression in peri-tumoral liver tissues of rats at different stages of carcinogen-induced hepatocarcinogenesis. *F*, senescence-associated β-galactosidase (SA-β-gal) staining to assess the level of cellular senescence in peri-tumoral liver tissues of rats at different stages of carcinogen induction. *G* and *H*, Immunohistochemical staining of CD133 and SOX-9 expression in peri-tumoral the liver tissues at 0 weeks (low senescence) and 12 weeks (high senescence) to evaluate the activation of hepatic progenitor cells (HPCs). P16, cyclin-dependent kinase inhibitor 2A; P21, cyclin-dependent kinase inhibitor 1A; SA-β-gal, senescence-associated β-galactosidase.

We found there were significant differences in the cellular senescence pathway by gene chip analysis and KEGG pathway enrichment analysis (Fig. 3*A*) and there was marked reduction in senescence-related genes in the AAV8-*Tmbim1* group (Fig. 3*B*). To further validate the relationship between TMBIM1 and senescence, we performed immunohistochemical staining of cancerous tissue from patients with liver cancer and the statistical analyses revealed the negative correlation between TMBIM1 and senescence markers cyclin-dependent kinase inhibitor 1A (P21) and cyclin-dependent kinase inhibitor 2A (P16) (Fig. 3*C*). Similarly, immunohistochemical staining of cancerous tissue from rats at 8 W and 12 W also showed a negative correlation between TMBIM1 and the senescence marker P16 (Fig. 3*D*). Increasing evidence suggests that CS is a critical factor for the occurrence of HCC. To verify the involvement of senescence in liver cancer progression, we established a primary liver cancer model (Fig. S2*A*) and the successful establishment was verified by liver morphology and H&E (Fig. S2, *B* and *C*). We observed a gradual increase in senescent cells with carcinogenesis progressed (Figs. 3*F* and S2*C*). Furthermore, we extracted RNA and protein from rat liver tissue for PCR and Western blot analysis, which showed mRNA expression levels of *Ckdn1a* and *Ckdn2a* increased (Fig. S2*D*) and the protein expression also increased (Fig. 3*E*). Additionally, IF staining of human cancerous tissue demonstrated colocalization of senescent cells and hepatocytes (Fig. S2*E*). Our previous research has indicated that HPCs promote HCC. We performed immunostaining of tissue sections at 0 W and 12 W that revealed the increase in senescent cells and the proliferation of HPCs marked by CD133 and CK19 (Figs. 3, *G*, *H* and S4, *D*, *E*), which further supports the notion that senescent cells can enhance HPC generation, thereby facilitating the occurrence of liver cancer. In summary, we found a negative correlation between TMBIM1 and senescence and during HCC occurrence, senescent cells promoted liver cancer progression by inducing the activation of HPCs.

### TMBIM1 inhibits CS to prevent HCC in rats

**Figure 4:**
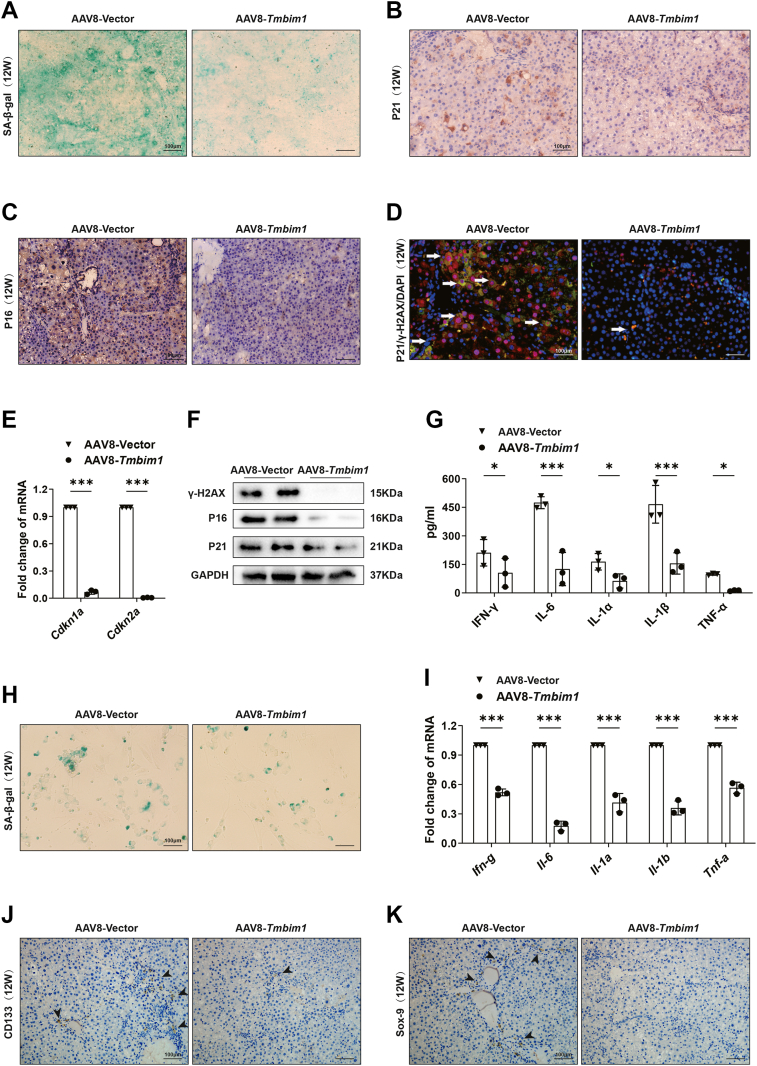
**TMBIM1 suppresses cellular senescence during hepatocellular carcinoma progression.***A*, SA-β-gal staining was used to assess the level of senescent cells in peri-tumoral liver tissues of rats overexpressing TMBIM1 at 12 weeks after carcinogen induction. *B* and *C*, immunohistochemical staining was performed to evaluate the expression levels of senescence-associated markers P21 and P16 in peri-tumoral liver tissues of rats overexpressing TMBIM1 at 12 weeks after carcinogen induction. *D*, IF analysis of colocalization between senescent cells and DNA damage in peri-tumoral liver tissues of rats overexpressing TMBIM1 at 12 weeks after carcinogen induction. IF staining shows P21-positive cells (*red*) and γ-H2AX-positive cells (*green*); nuclei were counterstained with DAPI (*blue*). *E*, qPCR analysis of the relative mRNA levels of Cdkn2a and Cdkn1a in liver tissues at 12 weeks after carcinogen induction. *F*, Western blot analysis of P16, P21, and γ-H2AX protein expression levels in liver tissues at 12 weeks after carcinogen induction. *G*, ELISA quantification of SASP factors (interleukin-1α, interleukin-1β, interleukin-6 (IL-6), interferon-γ (IFN-γ), tumor necrosis factor-α (TNF-α)) in rat serum at 12 weeks after carcinogen induction. *H*, SA-β-gal staining was used to detect the level of senescent cells in primary cells isolated at 12 weeks after carcinogen induction. *I*, qPCR analysis of the mRNA expression levels of SASP-related factors in primary cells at 12 weeks after carcinogen induction. *J and K*, Immunohistochemical staining was performed to evaluate the expression levels of CD133 and SOX-9 in peri-tumoral liver tissues of rats at 12 weeks after carcinogen induction, reflecting the activation status of hepatic progenitor cells (HPCs). All data are presented as mean ± SD (n = 3). ∗*p* < 0.05, ∗∗∗*p* < 0.001. IL-6, interleukin-6; P16, cyclin-dependent kinase inhibitor 2A; SA-β-gal, senescence-associated β-galactosidase; TNF-α, tumor necrosis factor-α.

In *Tmbim1*-overexpressed liver cancer rat models, β-galactosidase staining of frozen sections and immunohistochemical staining of paraffin-embedded sections of liver from rat at 12 W showed fewer senescent cells (Figs. 4, *A*–*C* and S4, *F*–*H*). Senescence is accompanied by DNA damage. Additionally, the co-localization of P21 and DNA damage marker γ-H2AX detected by IF assays was less than the control group (Figs. 4*D* and S4*I*). The mRNA expression levels of *Cdkn2a* and *Cdkn1a* in the livers of rats at post-carcinogenesis week 12 decreased significantly (Fig. 4*E*), which was consistent with the protein expression of P16, P21 and γ-H2AX (Fig. 4*F*). Senescent cells can secrete SASP but TMBIM1 reduced the quantity of SASP factors (IFN-γ, IL-6, interleukin-1α, IL-1β and TNF-α) (Fig. 4*G*). These results indicate that TMBIM1 inhibits cellular senescence during the progression of liver cancer. To further validate this result, we isolated primary liver cells from rats at week 12 post-carcinogenesis and found that the number of senescent cells decreased significantly in the AAV8-*Tmbim1* group compared to the control group by senescence-associated β-galactosidase (SA-β-gal) staining (Figs. 4*H* and S4*J*). The mRNA levels of SASP in primary liver cells also decreased (Fig. 4*I*). Previous studies have shown that senescent cells promote liver cancer by activating the HPCs. We performed histochemical of liver sections from rats and found that the number of HPCs marked by CD133 and SOX-9 reduced compared to the control group (Figs. 4, *J*, *K* and S4, *K*, *L*). Furthermore, we pretreated TMBIM1-overexpressing hepatocytes with LPS to specifically induce cellular senescence *in vitro*. These senescent TMBIM1-overexpressing cells (or nonsenescent controls) were then co-inoculated subcutaneously with tumor cells into nude mice. We observed that cellular senescence reversed the anti-tumor effects of TMBIM1 (Fig. S5, *F* and *G*). These results suggested that TMBIM1 inhibited the occurrence of liver cancer by suppressing CS.

### TMBIM1 inhibits LPS-induced hepatocyte senescence

**Figure 5:**
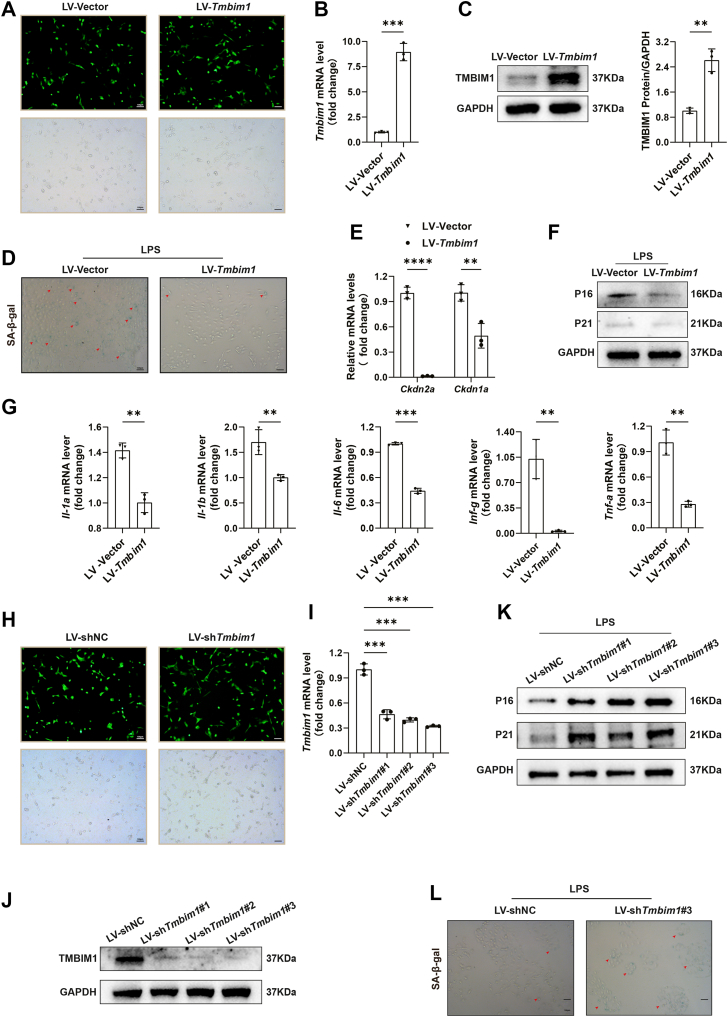
**TMBIM1 suppresses LPS-induced senescence in rat hepatocytes (BRL) *in vitro*.***A*, rat hepatocyte cell line infected with lentivirus overexpressing TMBIM1. Green fluorescence (*top*) and bright field (*bottom*) images are shown after infection. *B*, qPCR analysis of relative Tmbim1 mRNA levels after lentiviral infection.*C*, Western blot analysis of TMBIM1 protein expression after lentiviral infection. *D*, SA-β-gal staining detecting cellular senescence in lentivirus-infected hepatocytes treated with lipopolysaccharide (LPS) (50 ng/ml, 6 days). Senescent cells are shown in *blue*. *E*, qPCR analysis of relative Cdkn2a and Cdkn1a mRNA levels in lentivirus-infected hepatocytes treated with LPS (50 ng/ml, 6 days). *F*, Western blot analysis of P16 and P21 protein expression in lentivirus-infected hepatocytes treated with LPS (50 ng/ml, 6 days). *G*, qPCR analysis of relative mRNA levels of senescence-associated secretory phenotype factors (*Il-1a*, *Il-1b*, *Il-6*, *Ifn-γ*, *Tnf-α*) in lentivirus-infected hepatocytes treated with LPS (50 ng/ml, 6 days). *H*, rat hepatocyte cell line infected with lentivirus knocking down TMBIM1. Green fluorescence (*top*) and bright field (*bottom*) images are shown after infection. *I*, qPCR analysis of relative Tmbim1 mRNA levels after lentiviral knockdown infection. *J*, Western blot analysis of TMBIM1 protein expression after lentiviral knockdown infection. *K*, Western blot analysis of P16 and P21 protein expression in lentiviral knockdown-infected hepatocytes treated with LPS (50 ng/ml, 6 days). *L*, SA-β-gal staining detecting cellular senescence in lentiviral knockdown-infected hepatocytes treated with LPS (50 ng/ml, 6 days). Senescent cells are shown in blue. All data are presented as mean ± SD (n = 3). ∗∗*p* < 0.01, ∗∗∗*p* < 0.001, ∗∗∗∗*p* < 0.0001. ns, not significant. IL-6, interleukin-6; LPS, lipopolysaccharide; P16, cyclin-dependent kinase inhibitor 2A; SA-β-gal, senescence-associated β-galactosidase; SASP, senescence-associated secretory phenotype; TNF-α, tumor necrosis factor-α.

To further validate the role of TMBIM1 in the inhibition of CS, we carried out *in vitro* experiments. Rat liver BRL cells were treated with LPS (50 ng/ml) for 6 days to establish cellular senescence model. After LPS treatment, the number of senescent cells increased significantly (Fig. S3*A*). Additionally, the mRNA expression levels of *Cdkn2a*, *Cdkn1a* and *Tmbim1* decreased significantly compared to the control group and protein expression was also consistent with this downregulation (Fig. S3, *B* and *C*). Besides, the mRNA expression levels of SASP markers (*Il-1a*, *Il-1b*, *Il-6*, *Ifn-g* and *Tnf-a*) increased (Fig. S3*D*), which confirmed the successful establishment of LPS-induced senescence. After 48 h of infection of lentivirus to overexpress *Tmbim1* in the hepatocytes (LV-*Tmbim1* group), cells displaying viral fluorescence were observed under a microscope with nearly 100% of the cells exhibiting the expected phenotype (Fig. 5*A*). The mRNA levels of *Tmbim1* and the protein expression levels increased significantly (Fig. 5, *B* and *C*). Subsequently, we treated *Tmbim1*-overexpresed hepatocytes with LPS and the number of senescent cells reduced in the LV-*Tmbim1* group (Fig. 5*D*). Additionally, the mRNA expression levels of *Cdkn2a* and *Cdkn1a* and protein expression levels reduced (Fig. 5, *E* and *F*). The mRNA expression levels of SASP markers also decreased (Fig. 5*G*). Similarly, we knocked down TMBIM1 (LV-sh*Tmbim1* group) and IF confirmed successful retransmission (Fig. 5*H*). The mRNA expression of *Tmbim1* and protein levels reduced significantly (Fig. 5, *I* and *J*). Additionally, the protein expression of P16 and P21 was higher (Fig. 5*K*). The number of senescent cells was more in LV-*Tmbim1* group after LPS treatment (Fig. 5*L*). Collectively, these results demonstrated that TMBIM1 inhibited LPS-induced cellular senescence.

### TMBIM1 inhibits the activity of Smad2/3 in the TGF-β signaling pathway

**Figure 6:**
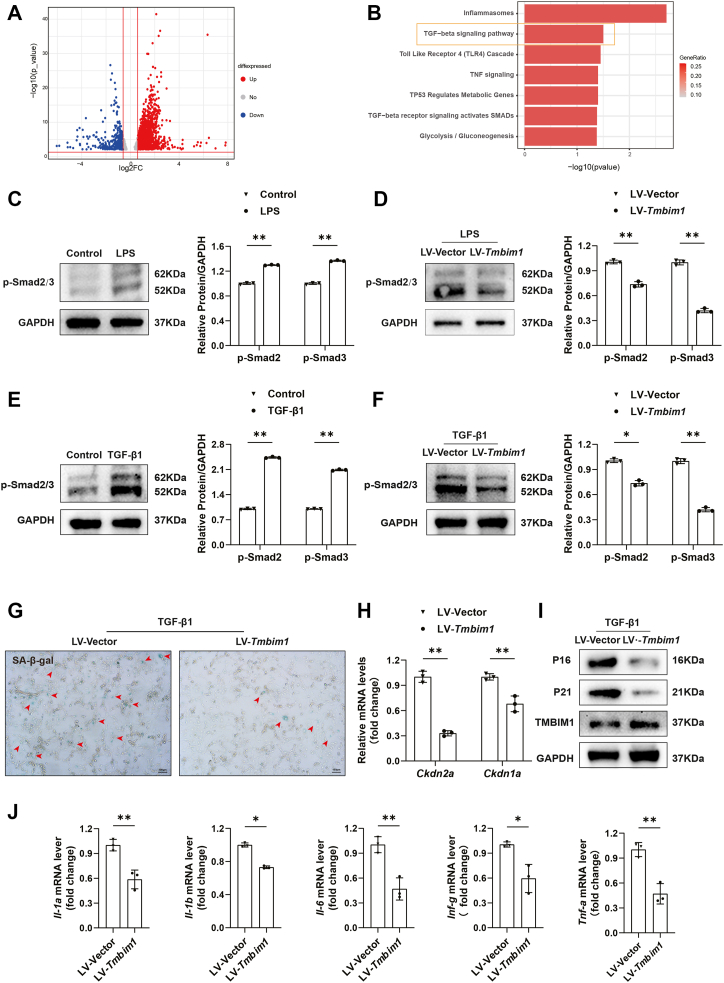
**TMBIM1 suppresses cellular senescence by inhibiting the TGF-β signaling pathway.***A*, microarray analysis of liver tissues from control and AAV8-Tmbim1 groups at 12 weeks after carcinogen induction. A volcano plot displays differentially expressed genes between the two groups. *B*, KEGG pathway enrichment analysis of differentially expressed genes between the two groups. *C*, Western blot analysis of p-Smad2/3 protein expression in hepatocytes treated with LPS to induce senescence. *D*, Western blot analysis of p-Smad2/3 protein expression in TMBIM1-overexpressing hepatocytes treated with LPS. *E*, Western blot analysis of p-Smad2/3 protein expression in hepatocytes treated with transforming growth factor beta 1 (TGF-β1) to induce senescence. *F*, Western blot analysis of p-Smad2/3 protein expression in TMBIM1-overexpressing hepatocytes treated with TGF-β1. *G*, SA-β-gal staining detecting senescent cells (*blue*) in TMBIM1-overexpressing cells treated with TGF-β1. *H*, qPCR analysis of relative Cdkn2a and Cdkn1a mRNA levels in TMBIM1-overexpressing cells treated with TGF-β1. *I*, Western blot analysis of P16 and P21 protein expression in TMBIM1-overexpressing cells treated with TGF-β1. *J*, qPCR analysis of relative mRNA levels of SASP-related factors (*Il-1a*, *Il-1b*, *Il-6*, *Ifn-γ*, *Tnf-α*) in TMBIM1-overexpressing cells treated with TGF-β1. All data are presented as mean ± SD (n = 3). ∗*p* < 0.05, ∗∗*p* < 0.01. IL-6, interleukin-6; P16, cyclin-dependent kinase inhibitor 2A; SA-β-gal, senescence-associated β-galactosidase; TGF-β1, transforming growth factor beta 1; TNF-α, tumor necrosis factor-α.

To investigate the effect of TMBIM1 on cellular senescence, differential gene analysis was conducted at 12 W and KEGG pathway enrichment analysis revealed significant differences in the TGF-β signaling pathway between control group and AAV8-*Tmbim1* group (Fig. 6, *A* and *B*). Increasing evidence indicates links between TGF-β signaling and age-related diseases (16). TGF-β signal transduction activates downstream Smad2/3 pathways through phosphorylation. We treated rat hepatocytes with LPS for 6 days and found that the TGF-β signaling pathway was activated; increasing p-Smad2/3 protein levels (Fig. 6*C*). *Tmbim1*-overexpresed cells also showed decreased p-Smad2/3 protein expression (Fig. 6*D*). These results suggested that LPS can activate the TGF-β signaling pathway, whereas TMBIM1 can inhibit LPS-induced activation of this pathway.

To verify that TMBIM1 suppresses cellular senescence through the TGF-β signaling pathway, we treated rat hepatocyte lines with 4-day interference of 20 ng/ml transforming growth factor beta 1 (TGF-β1), an activator of the TGF-β signaling pathway), leading to the activation of the pathway and a significant increase in p-Smad2/3 protein levels (Fig. 6*E*). Following TGF-β1 treatment, cellular senescence increased, as indicated by a significant increase in the number of blue SA-β-gal-positive cells (Fig. S3*E*). The mRNA and protein expression levels of *Cdkn1a* and *Cdkn2a* significantly increased (Fig. S3, *F* and *G*) and SASP was also significantly upregulated (Fig. S3*H*), indicating that TGF-β1 promotes cellular senescence by activating the TGF-β signaling pathway.

We found that p-Smad2/3 levels decreased significantly after TGF-β1 treatment of *Tmbim1*-overexpressed cells (Fig. 6*F*). After TGF-β1 treatment, the CS level in the *Tmbim1*-overexpressed group decreased, with a significant reduction in the number of blue SA-β-gal-positive cells in the LV-*Tmbim1* group (Fig. 6*G*). mRNA and protein expression levels of *Cdkn1a* and *Cdkn2a* were significantly decreased (Figs. 6, *H*, *I* and S4*Q*) and SASP was also significantly downregulated (Fig. 6*J*). Additionally, when TGF-β1 was applied to *Tmbim1* knockdown cells, an increase in CS was observed after treatment (Fig. S3, *I*–*K*). These results suggested that TMBIM1 inhibited CS in rat hepatocytes by blocking the TGF-β signaling pathway.

### TMBIM1 regulates the lysosomal degradation of TGFBR1

**Figure 7:**
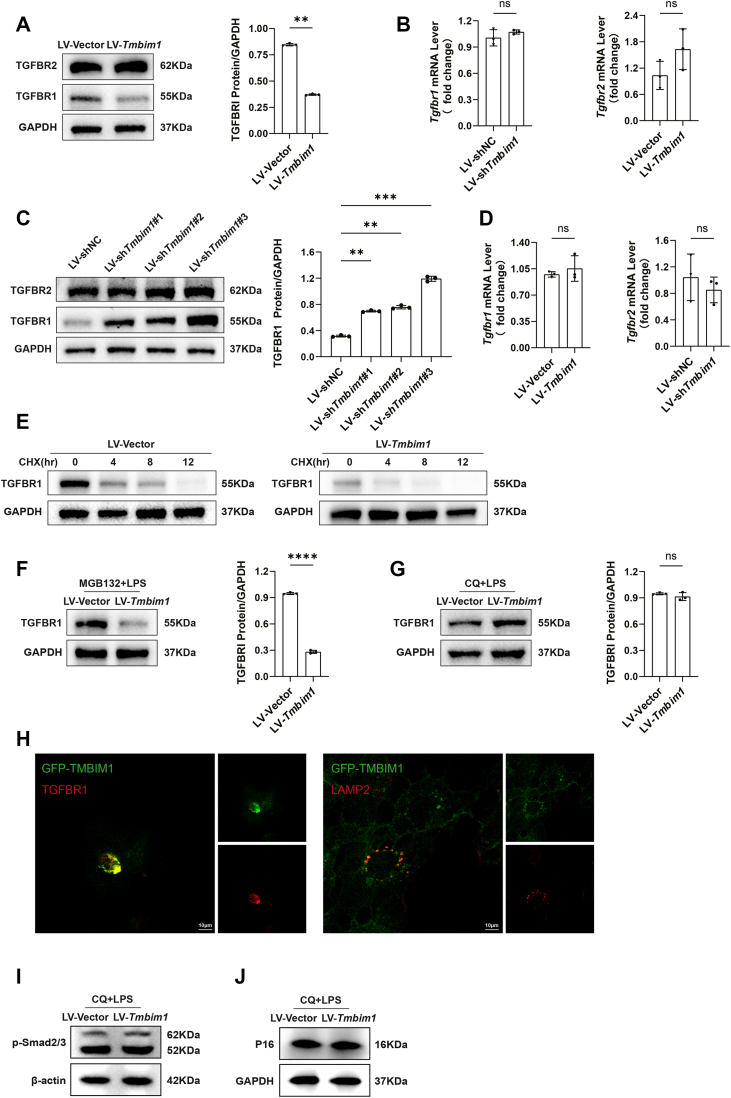
**TMBIM1 regulates the lysosomal degradation of TGFBR1.***A*, Western blot analysis of transmembrane BAX inhibitor motif containing 1 (TGFBR1) and TGF-β type II receptor (TGFBR2) protein expression in TMBIM1-overexpressing cells with LPS-induced senescence. *B*, qPCR analysis of Tgfbr1 and Tgfbr2 mRNA levels in TMBIM1-overexpressing cells with LPS-induced senescence. *C*, Western blot analysis of TGFBR1 and TGFBR2 protein expression in TMBIM1-knockdown cells with LPS-induced senescence. *D*, qPCR analysis of Tgfbr1 and Tgfbr2 mRNA levels in TMBIM1-knockdown cells with LPS-induced senescence. *E*, Western blot analysis of TGFBR1 protein expression in TMBIM1-overexpressing cells treated with the translation inhibitor cycloheximide. *F*, Western blot analysis of TGFBR1 protein expression in TMBIM1-overexpressing cells treated with the proteasome inhibitor MG132. *G*, Western blot analysis of TGFBR1 protein expression in TMBIM1-overexpressing cells treated with the lysosome inhibitor chloroquine (CQ). *H*, confocal microscopy images showing co-localization of GFP-TMBIM1 (*green*) with TGFBR1 and LAMP2 (*red*) in BRL cells infected with lentivirus expressing GFP-TMBIM1, as detected by IF. *I*, Western blot analysis of senescence markers P16 and P21 in cells treated with CQ and LPS. *J*, SA-β-gal staining detecting senescent cells (*blu*e) in cells treated with CQ and LPS. All data are presented as mean ± SD (n = 3). ∗∗*p* < 0.01, ∗∗∗*p* < 0.001, ∗∗∗∗*p* < 0.0001. ns, not significant. CQ, chloroquine; P16, cyclin-dependent kinase inhibitor 2A; SA-β-gal, senescence-associated β-galactosidase; TGFBR1, transmembrane BAX inhibitor motif containing 1; TGFBR2, TGF-β type II receptor.

TGF-β signaling primarily functions through type I and type II receptors, with TGFBR1 serving as a key receptor that transmits extracellular stimuli to downstream signaling pathways. Degradation of this receptor occurs *via* two pathways: proteasome-dependent and lysosome-mediated (17, 18). Lysosomal degradation is a crucial pathway for the regulation of TGF-β signaling. TMBIM1, a membrane protein of endosomes and lysosomes, is hypothesized to facilitate lysosomal degradation of TGFBR1, thereby influencing CS. Our experiments revealed *Tmbim1* only affect the TGFBR1 protein expression (Fig. 7, *A* and *C*) but the mRNA levels of *Tgfbr1* and *Tgfbr2* were unaffected (Fig. 7, *B* and *D*). Considering the post-translational modifications and degradation of TGFBR1 are stringently regulated and essential for the activity of the TGF-β signaling pathway, we evaluated the stability of TGFBR1 in both control and LV-*Tmbim1* groups following treatment with the translational inhibitor cycloheximide. In the *Tmbim1*-overexpressed group, TGFBR1 was degraded (Fig. 7*E*). To elucidate the pathway through which TMBIM1 degrades TGFBR1, we pretreated cells with the broad-spectrum proteasome inhibitor (MGB132) and lysosomal inhibitor chloroquine (CQ). MGB132 did not reverse the reduction in TGFBR1 expression in *Tmbim1*-overexpressed BRL cells (Fig. 7*F*), whereas CQ reversed this decrease in TGFBR1 expression in these cells (Fig. 7*G*). These results confirmed that TMBIM1 increases TGFBR1 degradation *via* a lysosome-associated pathway. To further verify this study, we transfected liver cells with a TMBIM1-GFP (green fluorescent protein) fusion construct. IF staining was performed using primary antibodies against TGFBR1 and LAMP2 to label TGFBR1 and the lysosomes. Confocal microscopy analysis demonstrated a significant colocalization of TMBIM1 with both lysosomal markers and TGFBR1, further indicating that TMBIM1 degrades TGFBR1 through the lysosomal pathway. In addition, TMBIM1-overexpressing hepatocytes were treated with CQ and LPS. CQ treatment abrogated the downregulation of TGFBR1 induced by TMBIM1 overexpression. Subsequently, we observed that activation of the TGF-β signaling pathway, as reflected by p-Smad2/3 levels (Fig. 7*I*), was restored. Concurrently, the expression of senescence markers P16, along with SA-β-gal activity, were markedly increased (Fig. 7*J*). These results suggest that TMBIM1 promotes TGFBR1 degradation by regulating the lysosomal pathways to inhibit cellular senescence.

### TMBIM1 interacts with the Rab9 protein

**Figure 8:**
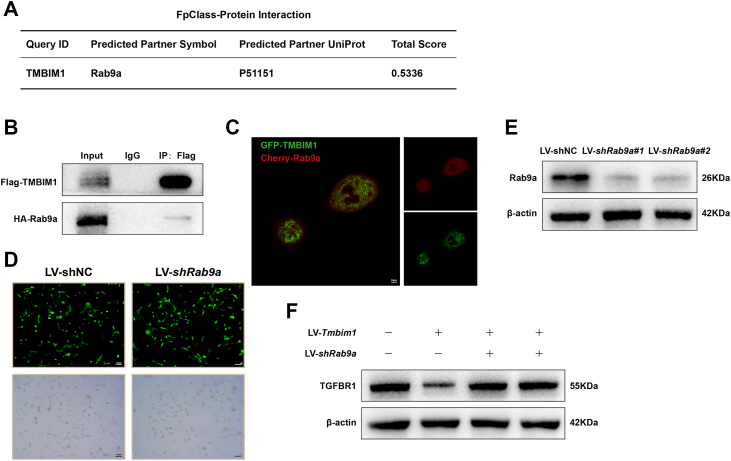
**TMBIM1 may interact with Rab9a.***A*, A protein-protein interaction prediction website indicated a high probability of interaction between TMBIM1 and Rab9a (http://dcv.uhnres.utoronto.ca/FPCLASS/ppis/). *B*, Coimmunoprecipitation assay in BRL cells co-expressing Flag-Tmbim1 and HA-Rab9a fusion proteins *via* lentiviral vectors. Cell lysates and immunoprecipitates were analyzed by Western blot using anti-Flag and anti-HA antibodies, with IgG as a negative control. *C*, Confocal microscopy images showing colocalization of GFP-Tmbim1 (*green*) and Cherry-Rab9a (*red*) fluorescent fusion proteins in BRL cells infected with lentiviral vectors. *D*, hepatocytes co-infected with lentiviruses; *green* fluorescence (*top*) and bright field (*bottom*) images are shown under a fluorescence microscope. *E*, Western blot analysis of Rab9a protein expression in cells after Rab9a knockdown. *F*, Western blot analysis of TGFBR1 protein expression in Rab9a-knockdown cells overexpressing TMBIM1.

Research has shown that Rab GTPase proteins play a crucial role in the signal transduction process of the TGF-β signaling pathway. Specifically, Rab5 mediates the fusion of endocytic vesicles to form early endosomes, Rab7 is responsible for transport between late endosomes and lysosomes and Rab9a is involved in the fusion of endosomes and lysosomes, as well as transport from late endosomes to the trans-Golgi network (19, 20, 21). To preliminarily analyze whether TMBIM1 can interact with Rab proteins, we first consulted a protein interaction prediction website (http://dcv.uhnres.utoronto.ca/FPCLASS/ppis/) and found a high probability of interaction between TMBIM1 and Rab9a (Fig. 8*A*). To validate these results, Flag-*Tmbim1* and HA-*Rab9a* fusion protein-expressing lentiviruses were constructed. After expressing these proteins in BRL cells, we performed co-immunoprecipitation experiments and found that Flag precipitated HA-Rab9a (Fig. 8*B*). Additionally, BRL cells were simultaneously infected with GFP-TMBIM1 and Cherry-Rab9a fusion fluorescent protein lentiviruses and confocal microscopy images showed that TMBIM1 and Rab9a co-localized (Fig. 8*C*). Subsequently, we infected LV-*Tmbim1* cells with a *Rab9a*-knockdown lentiviral and observed that the downregulation of *Rab9a* could reverse the degradation of TGFBR1 by *Tmbim1* (Fig. 8*D*), The interaction between Tmbim1 and Rab9a may promote the degradation of TGFBR1 to suppress the TGFBR1 signaling pathway, but the specific mechanism requires further validation. Finally, we knocked down Rab9a in mice using adenoviral transduction (Fig. S5*A*) and then orthotopically implanted tumor cells into the liver. We observed that Rab9a knockdown alone significantly promoted tumor growth. Moreover, Rab9a downregulation reversed the tumor-suppressive effect of Tmbim1 (Fig. S5, *B*–*E*), indicating that Rab9a is essential for TMBIM1-mediated suppression of tumor progression through the proposed mechanism.

## Discussion

HCC is a highly lethal malignant tumor. Although various clinical treatment options are available, the prognosis remains poor, with a high recurrence rate and significant resistance to therapies and malignant metastasis is difficult to control (22, 23). We have identified that the expression of TMBIM1 is higher in the adjacent nontumor tissues of HCC patients than in the tumor tissues and gradually decreases in DEN-induced rat models. Additionally, overexpressed of TMBIM1 inhibited the formation of tumor nodules and reduced tumor size during DEN-induced liver cancer progression in rats. These findings suggested that TMBIM1 plays a role in suppressing the occurrence of liver cancer. Interestingly, at the ninth week of carcinogen-induced hepatocarcinogenesis, we administered adenoviruses carrying TMBIM1 knockdown or overexpression constructs *via* tail vein injection in rats. Tumor development was monitored until week 14, a stage corresponding to the progression phase of liver cancer. Strikingly, TMBIM1 overexpression unexpectedly enhanced tumor invasiveness and promoted tumor growth (Fig. S5, *F*-*L*). These results demonstrate a dual role of TMBIM1 in hepatocellular carcinoma. While this apparent paradox is complex, it represents a well-recognized phenomenon in cancer biology. Based on our findings, we hypothesize that TMBIM1 may facilitate tumor progression and malignancy by suppressing cellular senescence in tumor cells, a possibility that has not been further explored in the present study.

An increasing body of research indicates the important effects of hepatocyte senescence on chronic liver diseases (24). In a mouse liver cancer model induced by DEN-CCl_4_, an increase in the number of senescent hepatocytes was observed during the induction of hepatocellular carcinoma and senescent cells promoted the occurrence of liver cancer by secreting SASP and activating macrophages (25). Another study revealed a temporal relationship between CS and HCC, indicating that the progression of liver cancer is accompanied by an increase in CS (26). Similarly, in our study, we observed a gradual increase in CS during the occurrence of HCC in a rat DEN-induced liver cancer model. *Tmbim1* overexpressed inhibited the occurrence of CS. Immunohistochemical, Western blot and PCR results demonstrated a significant reduction in the senescence markers *Cdkn2a* and *Ckdn1a* and a notable decrease in SASP in the *Tmbim1* overexpressed groups. Additionally, substantial evidence indicates that SASP can create a microenvironment conducive to cancer occurrence, which has been shown to induce the promotion, progression and metastasis of cancer stemming from tumor cells of various tissues and origins, including the nervous system, breast, skin, stomach, oral cavity, prostate and ovaries (27). Studies have shown that TMBIM1 is widely expressed in various tissues and organs (9). Our findings confirmed that the expression of TMBIM1 on the membranes of late endosomes and lysosomes significantly reduced SASP, suppressed CS and inhibited the occurrence of HCC, thereby filling a gap in the role of CS and HCC and providing insights into the impact of TMBIM1 on other tumors.

In patients with chronic liver disease/cirrhosis, the ability to detoxicate, biodegrade and clear LPS and other bacterial products is impaired. The overgrowth and gene mutation of intestinal bacteria and the condition of increased intestinal permeability circulate LPS from the intestine to the liver through the portal vein (28, 29). With increasing serum LPS levels, there are greater quantities of *Escherichia coli* and other gram-negative bacteria in the gut microbiome (30). Additionally, translocation of LPS to the liver is associated with persistently low levels of LPS in the liver (31). LPS can induce CS and has been frequently used as an inducer in CS models (32). However, the relationship between LPS and aging remains incompletely understood. We established LPS-induced rat BRL senescent cell models to investigate the mechanism by which TMBIM1 inhibits hepatocarcinogenesis and found that TMBIM1 can suppress CS by activating the TGF-β signaling pathway.

To date, research on TMBIM1 has been mainly associated with inflammation and apoptosis, however, no studies have been conducted on CS. Previous study has found that TMBIM1 regulates vascular remodeling by inhibiting the production of MMP-9 (10, 33). MMP-9 modulates senescence-associated inflammation to alleviate age-related cardiac fibrosis and diastolic dysfunction, induces the accumulation of ventricular macrophages, promotes macrophage polarization and results in cardiac aging and inflammation (34). In skin aging models using cigarette irradiation and lung aging models, the expression levels of senescence-associated genes (*Cdkn2a* and *Cdkn1a*) and *Mmp-9*, which are SASP markers, increased markedly (35, 36). Additionally, in vitro-induced CS studies have shown that enhancing the secretion of MMP-9 can promote nasopharyngeal carcinoma progression of nasopharyngeal carcinoma (37). However, these findings suggest an obscure relationship between TMBIM1 and CS. Fortunately, our research has provided direct evidence that TMBIM1 can inhibit CS, revealing a new function of TMBIM1 and given light to further research on the relationship between TMBIM1, CS and other diseases.

We found that TMBIM1 interacts with Rab9a, localizing within lysosomes, which promotes the degradation of TGFBR1 and inhibits the TGF-β signaling pathway, thereby reducing CS and preventing HCC. Consequently, targeting lysosomal regulatory factors to control protein degradation is a promising approach for liver cancer treatment. This strategy might also assist in developing therapeutic strategies for other age-related diseases. Furthermore, TMBIM1 is a membrane protein located on the nuclear endosome and lysosomal membranes, facilitating lysosomal degradation of TGFBR1 through its interaction with Rab9a. It is worth investigating whether this mechanism is applicable to the recycling of other membrane receptors under various pathological conditions. The TMBIM1 family comprises six highly conserved members that are involved in various biological processes. Future studies should determine if other family members have similar effects.

In summary, we have identified TMBIM1 as a significant negative regulator of liver cancer in rats. We discovered that TMBIM1 exerts inhibitory effects on CS, revealing the molecular mechanism by which TMBIM1 suppresses liver cancer progression. Additionally, CS is associated with various age-related diseases; clearance of senescent cells or blockade of their effects may delay the onset of aging-related conditions and anti-aging pharmacological treatments have shown promise in osteoporosis management (38, 39). Senescence occurs in the early stages of acute kidney injury and is critical for prognosis, while chronic senescence plays a dominant role in the progression of acute kidney injury to chronic kidney disease (40). Some approaches targeting CS, such as senolytics and senomorphics, have emerged as potential treatments for asthma (41, 42). Furthermore, CS is linked to other diseases such as biliary injury, atherosclerosis and macular degeneration (43, 44, 45). Our study showed that TMBIM1 regulates lysosomal pathways to promote the degradation of TGF-β type I receptors, suppress CS and inhibit HCC, thus providing a novel therapeutic strategy for preventing liver cancer and may shed light on other senescence-related diseases.

## Experimental procedures

### Animals

In this experiment, 10-week-old male wild-type SD rats (120 ± 20 g) were purchased from Shanghai Jihui Experimental Animal Care Co., Ltd. The rats were fed purified water containing diethylnitrosamine (DEN) solution at 0, 4, 8, 12 and 14 weeks to establish a primary liver cancer model. Furthermore, to establish models of both Tmbim1-overexpressing hepatocarcinogenesis and subsequent cancer progression, AAV8-Tmbim1 was delivered *via* tail vein injection at the second and ninth weeks, respectively, after the initiation of DEN-induced carcinogenesis, with continued DEN feeding thereafter. All animal experimental protocols were approved by the Institutional Animal Care and Use Committee of the Institute of Health Sciences. All animals received humane care according to the criteria outlined in the Guide for the Care and Use of Laboratory Animals.

To evaluate the suppressive role of TMBIM1 in hepatocarcinogenesis through the regulation of cellular senescence, a subcutaneous xenograft tumor model was established in male nude mice. Logarithmic-phase liver tumor cells were harvested and resuspended together with TMBIM1-overexpressing senescent hepatocytes in a mixture of PBS and Matrigel (1:1 ratio). A cell suspension containing tumor cells and senescent cells at a 1:2 ratio was subcutaneously injected into the flanks of the mice. Tumor growth was monitored periodically, and after 14 days, the mice were euthanized. Tumors were then excised, weighed, and processed for further analysis.

Liver tumor cells in the logarithmic growth phase were harvested and resuspended in a sterile Matrigel solution. Immunodeficient nude mice were anesthetized, and a small abdominal incision was made to exteriorize the left lobe of the liver. A cell suspension containing 1 × 10ˆ6 cells was slowly injected into the parenchyma of the left lateral lobe using a micro-syringe. To prevent leakage and ensure localized tumor growth, the injection site was gently pressed with a cotton swab for approximately 30 to 60 s after needle withdrawal. The liver lobe was then carefully returned to the abdominal cavity, and the incision was sutured. The mice were randomly assigned into the following groups: Rab9a-KO, Rab9a-KO combined with TMBIM1-overexpression , control, and TMBIM1-overexpression.

### Cell senescence models

LPS was diluted in serum-free high-glucose Dulbecco's Modified Eagle Medium (DMEM) to a final experimental concentration of 50 ng/ml. Cells were treated with 2 ml of the prepared LPS solution per well for a period of 6 days to induce cellular senescence.

TGF-β1 was diluted in serum-free high-glucose DMEM to achieve an experimental concentration of 20 ng/ml. Cells were treated with 2 ml of the prepared TGF-β1 solution per well for a duration of 4 days to elicit cellular senescence.

### Patient samples

Liver cancer and adjacent tissue specimens were collected from 70 patients with liver cancer who underwent liver resection at the Shanghai East Hospital of Hepatobiliary Surgery. The specimens were analyzed using histological and immunohistochemical staining techniques. Written informed consent was obtained from the patients before the study and the study was approved by the Ethics Committee of Shanghai East Hospital of Hepatobiliary Surgery. All studies involving human subjects were conducted in accordance with the principles set forth in the Declaration of Helsinki.

### Statistical analysis

All experimental data were analyzed using GraphPad Prism 8.0 (GraphPad Software; https://www.graphpad.com) for analysis of variance. Quantitative data from each experiment are expressed as mean ± SD and intergroup significance was assessed using the Student’s *t* test. Results from bioinformatic analyses, such as heat maps, were generated using the R language package. Clinical data analysis was performed using SPSS 22.0, employing Kaplan-Meier and Log-Rank analyses to compare survival rates among subgroups of patients. Statistical significance is defined as ∗*p* < 0.05, ∗∗*p* < 0.01, ∗∗∗*p* < 0.001 and ∗∗∗∗*p* < 0.0001.

### Histological analysis

Livers were fixed in 4% paraformaldehyde, paraffin-embedded and sectioned. H&E, mmunohistochemistry and IF staining was performed and each sample was evaluated and analyzed by a pathologist.

### SA-β-gal staining

SA-β-gal staining was performed using the β-galactosidase kit (Beyotime Biotechnology, C0602). Frozen sections of rat liver (hepatocyte cell line BRL) were washed once with PBS, added with β-galactosidase staining fixative, fixed for 15 min at room temperature, washed three times with PBS and then added with staining working solution (β-galactosidase staining solution A, β-galactosidase staining solution B, β-galactosidase staining solution C, X-Gal solution). Incubation was carried out overnight at 37 °C (in an incubator with no CO_2_ performed), senescent cells were observed under an ordinary microscope and three fields of view were randomly selected for counting statistics.

### Immunohistochemistry and immunofluorescence

Paraffin-embedded sections of tissue samples were subjected to immunohistochemistry and IF analysis. Immunohistochemistry was performed with primary antibodies including rabbit antibody TMBIM1 (dilution 1:50, NOVUS, NBP1-81310), rabbit antibody P16 (dilution 1:50, abcam, ab51243), rabbit antibody P21 (dilution 1:50, Cell Signaling #2947), rabbit antibody α-smooth muscle actin (dilution 1:500, Cell Signaling #19245S), rabbit antibody CD68 (dilution 1:200, abcam, ab31630), rabbit antibody caspase8 (dilution 1:200, Proteintech, 13423-1-AP) and rabbit antibody Fas/CD95 (dilution 1:200, Proteintech, 13098-1-AP). All of the antibodies were incubated overnight at 4 °C. Following 24-h incubation with the universal secondary antibody Supervision (d-3004), the samples were visualized by a microscope.

Immunofluorescence (IF) was performed with primary antibodies including rabbit antibody TMBIM1 (dilution 1: 50, NOVUS, NBP1-81310), rabbit antibody P16 (dilution 1: 100, abcam, ab51243), rabbit antibody P21 (dilution 1: 400, Cell Signaling, #2947), rabbit antibody hepatocyte nuclear factor (dilution 1:1000, abcam, ab201460), rabbit antibody γ-H2AX (dilution 1:5000, NOVUS, NB100-384) and they were incubated overnight at 4 °C. Subsequently, the TSA Plus Fluorescence Double Staining Kit (Servicebio, G1226-50T) was applied in strict accordance with the manufacturer's protocol.

### Immunocytochemistry

Immunocytochemistry (ICC) was performed with primary antibodies including mouse antibody TGFBR1 (dilution 1:100, Affinity, BF8256) and rabbit anti-LAMP2 (dilution 1:500, sigma, L0668) and they were incubated at 4 °C overnight. Subsequently, the samples were incubated with a red-fluorescent secondary antibody, Alexa Fluor 568 goat anti-rabbit (Life Technologies, catalog number A11011), to enable the visualization.

### Quantitative PCR

Total RNA was extracted from tissues and cells with the use of Trizol (Beckman Coulter, G3013) and reverse transcribed into cDNA by Bestar qPCR RT Mix (gDNA Remover) (DBI Bioscience, DBI-2220) kit. PCR amplification was performed with the use of SYBR Green. Products were quantified by PCR conditions (95 °C 10 s, 60 °C 30 s, 72 °C 30 s, 40 cycles) to normalize the expression level of targeted gene mRNA. The primer sequences for this study are shown in Table S1.

### Western blot

Total proteins were isolated by treating tissues and cells with RIPA (Servicebio, G2002) lysis buffer containing PMSF (Servicebio, G2008). The different proteins were separated by electrophoresis with the use of a 10% SurePAGETM-15wells precast gel (Kingsley, M01215C) and the proteins were transferred to PVDF membranes (Millipore, IPVH00010) by transmembrane apparatus. The PVDF membrane was closed with a protein-free rapid closure solution (Servicebio, G2052), while the primary antibody was added and incubated at 4 °C overnight. The next day, the strips were washed by TBST and incubated with a secondary antibody for 1.5 h at room temperature and the signals were imaged with HRP Substrate peroxide solution (WBKLS0500) to develop the signal through the I. Finally, protein expression was quantified by ImageJ software (https://imagej.nih.gov/ij/) and normalized to GAPDH. In this experiment, antibodies specific to TMBIM1 (NOVUS, NBP1-81310), P16 (abcam, ab51243), P21 (Cell Signaling, #2947), γ-H2AX (NOVUS, NB100-384) and Smad2 (Cell Signaling, 5339T), Smad3 (Cell Signaling, #9523), p-Smad2/3 (Cell Signaling, 8828S), TGFBR1 (abcam, ab235578) and TGFBR2 (Affinity, AF5449) were used and all of these antibodies were diluted at 1:1000.

### Liver function tests and serum factor analysis

Serum samples from rats were collected by centrifuge at 3000 rpm for 10 min to obtain the supernatant and the serum concentrations of alanine aminotransferase and aspartate aminotransferase were detected by using an automatic biochemical analyzer (Shenzhen Radu Life Science and Technology, Chemray 800) to assess the liver function of the animals. The serum cytokine concentrations of interleukin-1α (ab235646), IFN-γ (ab239425), IL-1β (ab255730), IL-6 (ab234570), TNF-α (ab236712), IL-2 (ab221835), IL-4 (ab100770) and IL-13 (ab269547) in rats were detected by ELISA and the above Kit manufacturers were abcam.

### Primary cell isolation and culture

Hepatocytes were isolated from the rats’ livers of the Control and AAV8-*Tmbim1* groups, cultured in high sugar medium DMEM (Genpei, L110KJ) supplemented with 10% fetal bovine serum (Gibco,10437-028) and 1% penicillin-streptomycin-amphotericin B (Servicebio, G4015) and assayed at generation 3 to 6. Primary hepatocytes were isolated from the livers of 12-weeks DEN-induced male SD rats by collagenase perfusion and the tissues were digested by perfusing collagenase type IV solution (Servicebio, GC305015). The livers were removed, minced and passed through a 70 μm cell strainer (Biosharp, BS-100-SC) and the fluid obtained after filtration was centrifuged at 50 g to collect hepatocytes. The primary hepatocytes were then further isolated and purified with the use of Percoll's liquid (Biosharp, BS909) and the hepatocytes were inoculated in a DMEM medium.

### Microarray performance and analysis

To unravel the molecular mechanisms involved in the inhibition of hepatocarcinogenesis by TMBIM1, RNA was extracted from the livers of DEN-induced rats in AAV8-*Tmbim1* and Vector groups. Microarray analysis was performed on the rat samples and the gene expression levels were examined during the microarray analysis and the raw data were normalized by the robust multiple-array average. Differential genes were identified with the limma R package and the cutoff value was critical when the multiplicity was greater than 2, with significance at *p* < 0.05. The KEGG (http://www.kegg.jp) analysis was performed to analyze potential upstream regulators enriched for differential genes using the clusterProfiler R package—normalized expression of DEGs into heatmaps.

### Lentiviral vector construction and viral packaging

According to the experimental requirements, the corresponding vectors were selected and the PCR primers for the target fragment were designed. After digestion with the vector, agarose gel electrophoresis was performed to recover the target fragment, which was ligated with the vector and then extracted into a plasmid. Recombinant lentivirus was prepared by co-transfecting 293T cells with the targeted gene or shRNA plasmid packaged in lentiviral expression vectors psPAX2 and pMD2G. The lentiviral supernatant was collected at 48 h and 72 h of transfection and the lentiviral preservative was concentrated by ultracentrifugation to obtain high titer lentiviral preservative and finally the lentiviral indexes were determined according to the strict quality standards. Target vector information and designed primer information are shown in Table S2.

### Co-immunoprecipitation (CO-IP) assay

Rat hepatocyte cell lines (BRL) were infected with lentivirus (Flag-*Tmbim1*, HA-*Rab9a*) for 48 h and immune complexes were extracted from the cells with the use of the PierceTM Classic IP Kit (Thermo Fisher Scientific, 26146) kit according to the protocol provided by the manufacturer. Afterward, immunoblotting was performed with Flag (dilution 1:1000, Sigma, F1804) and HA (1:1000, Abmart, M20053L) primary antibodies, universal avoidance antibody (H + L) secondary antibody (1:1000, Abmart, M21008) and immunoblotting was performed with HRP Substrate peroxide solution (WBKLS0500) to visualize protein bands.

### Sirius red staining

After dewaxed and hydrated, paraffin sections were soaked in hematoxylin dye solution for 5 to 10 min and then washed with distilled water to remove the dye solution. A drop of Serina scarlet dyeing solution (Servicebio, GP1033) was infiltrated for 20 min. The sections were rinsed gently with running water later. After removing the staining solution, the slices are dehydrated and transparent with ethanol and sealed with neutral resin.

### Statistical analysis

Statistical analysis of the experimental data and the creation of bar graphs were conducted using GraphPad Prism 9 software. Quantitative data are presented as mean ± standard deviation (Mean ± SD). When comparing differences between two independent samples, if the data follow a normal distribution and meet the assumption of equal variances, a Student’s *t* test is employed. In cases where the data distribution is unclear or contains outliers, a more appropriate nonparametric test, such as the Mann-Whitney U test, is utilized. For comparisons among multiple independent samples, if the data meet the assumptions of normality and equal variances, one-way analysis of variance (One-way ANOVA) is applied. If the data do not satisfy the assumptions of normality and equal variances, the nonparametric Kruskal-Wallis H test is chosen. To analyze the degree of correlation between two samples, the Pearson method is used, and a *p*-value of less than 0.05 is considered statistically significant, indicating a difference.

## Data availability

Data for this study are available from the corresponding author upon request. All primers and other nucleic acid sequences are described in the Methods section.

## Supporting information

This article contains supporting information.

## Conflict of interest

The authors declare that they have no conflicts of interest with the contents of this article.
