# Supplementary material for: Transmembrane BAX inhibitor motif containing 1 inhibition of lysosomal degradation of TGF-β receptor 1 suppresses cellular senescence and hepatocarcinogenesis
Source: J Biol Chem. 2025 Nov 4;301(12):110904. doi: 10.1016/j.jbc.2025.110904 (PMC12702014; doi:10.1016/j.jbc.2025.110904)
Supplement: Supplementary Material [file mmc1.docx]

**Title page**

**TMBIM1 inhibits** **hepatocarcinogenesis by accelerating the** **lysosomal degradation of TGFBR1 to suppress cellular senescence**

**Daoyu Zhou（周道宇）^1,^** **^2^**^#^**,Wei Yu（俞薇）^1, 2^**^#^**, Yating Zheng（郑雅婷）^1^**^#^**,Xiaojuan Hou（侯晓娟）^2, 3^**^#^**, Kuizhi Zhang（张魁枝）^5, 1^, Xiaofeng Qian（钱晓峰）^6^, Lixia Duan（段丽霞）^7^, Shiyao Feng（冯诗尧）^8^, Mengmeng Xue（薛梦梦）^5, 1^, Xinyu Zhu（朱鑫宇）^1, 2^, Hengyan Zhang（张恒炎）^1^, Luyao Zhang（章璐瑶）^1^, Lixin Wei（卫立辛）^2, 3^, Wenting Liu（刘文婷）^4*^, Jinghua Jiang（姜京花）^2, 3*^,** **Li Zhang（张黎）^1, 5*^.**

^a^ Clinical Research Unit, The First Affiliated Hospital of Naval Medical University, Shanghai 200433, China

^b^ Tumor Immunology and Metabolism Center, National Center for Liver Cancer, Naval Medical University, Shanghai 201899, China

^c^ Department of Hepatic Surgery, Third Affiliated Hospital of Naval Medical University, Shanghai 201899, China

^d^ Department of Medical Oncology, Fudan University Shanghai Cancer Center, Shanghai 200433, China

^e^ Department of Clinical Pharmacology, Second Affiliated Hospital of Anhui Medical University, Hefei 230601, China

^f^ Shanghai Putuo District Liqun Hospital, Shanghai 200333, China

^g^ Department of Intensive Care Medicine, Shanghai Sixth People's Hospital, Shanghai 200233, China

^h^ Department of Urology, Chaohu Hospital of Anhui Medical University, Hefei 230601, China

*Corresponding authors:

E-mail addresses: [lizhangpaper@163.com](mailto:lizhangpaper@163.com) (L. Zhang). [wajjh1978@163.com](mailto:wajjh1978@163.com) (Jh. Jiang). [liuwenting1015@163.com](mailto:liuwenting1015@163.com) (Wt. Liu)

^#^Daoyu Zhou, Wei Yu, Yating Zheng and Xiaojuan Hou contributed equally to this paper.

**Supplementary material**

**Supplementary Figure 1. TMBIM1 suppresses hepatic fibrosis, inflammation, and apoptosis during hepatocarcinogenesis. (A)** Representative Sirius Red staining of liver sections from control and AAV8-Tmbim1-treated rats at 12 weeks post-carcinogen induction. Collagen deposition (red) indicates the extent of fibrosis. **(B)** Immunohistochemical analysis of α-smooth muscle actin (α-SMA) expression in liver sections from control and AAV8-Tmbim1 groups at 12 weeks post-carcinogen induction. **(C)** Serum levels of inflammatory cytokines measured by ELISA in control and AAV8-Tmbim1 groups at 12 weeks post-carcinogen induction. **(D)** Immunohistochemical detection of CD68 expression in liver sections from control and AAV8-Tmbim1 groups at 12 weeks post-carcinogen induction. **(E, F)** Immunohistochemical analysis of caspase-8 and Fas/CD95 expression in liver sections from control and AAV8-Tmbim1 groups at 12 weeks post-carcinogen induction. **(G)** TUNEL staining of liver sections from control and AAV8-Tmbim1 groups at 12 weeks post-carcinogen induction. Apoptotic cells are shown in green; nuclei are counterstained with DAPI (blue). All data are presented as mean ± SD (n=3). *p < 0.05, **p < 0.01, ***p < 0.001. ns, not significant.

**Supplementary Figure 2. Hepatic cellular senescence increases during hepatocarcinogenesis, and TMBIM1 promotes HCC progression. (A)** Schematic of the DEN-induced rat hepatocarcinogenesis model. Liver tissues were collected at different time points. **(B)** Representative macroscopic images of livers from rats at different stages of carcinogen induction. **(C)** HE staining (top) and immunohistochemical detection of P16 expression (bottom) in liver tissues at different stages of carcinogenesis. **(D)** qPCR analysis of Cdkn2a and Cdkn1a mRNA levels in liver tissues at different stages of carcinogenesis. **(E)** Immunofluorescence staining of human peri-tumoral liver tissues showing localization of hepatocytes and senescent cells. HNF-4α-positive cells (red) and P16/P21-positive cells (green) are shown; nuclei were counterstained with DAPI (blue). **(F)** Schematic of the experimental design: adenoviruses for TMBIM1 overexpression or knockdown were administered via tail vein injection at 9 weeks after DEN initiation to establish rat HCC models. **(G)** Macroscopic images of livers from different treatment groups at 14 weeks post-carcinogen induction. **(H)** Quantitative analysis of tumor numbers in livers of rats with TMBIM1 overexpression or knockdown. **(I)** Measurement of tumor sizes in livers of rats with TMBIM1 overexpression or knockdown. **(J)** HE staining of liver tissues from TMBIM1 overexpression or knockdown groups to assess pathological changes. **(K)** Survival analysis of rats after TMBIM1 overexpression or knockdown. **(L)** Body weights of rats in different treatment groups.

All data are presented as mean ± SD (n=3). *p < 0.05, **p < 0.01, ***p < 0.001.

**Supplementary Figure 3. Knockout of Tmbim1 promotes TGF-β-induced senescence in rat hepatocytes (BRL). (A)** SA-β-gal staining detecting senescent cells (blue) in BRL cells treated with LPS (50 ng/mL) for 6 days to establish a senescence model. **(B)** qPCR analysis of *Cdkn1a* and *Cdkn2a* mRNA levels in BRL cells after LPS treatment. **(C)** Western blot analysis of P16, P21, and TMBIM1 protein expression in BRL cells after LPS treatment. **(D)** qPCR analysis of relative mRNA levels of senescence-associated secretory phenotype (SASP) factors (*Il-1a*, *Il-1b*, *Il-6*, *Ifn-γ*, *Tnf-α*) in BRL cells after LPS treatment. **(E)** SA-β-gal staining detecting senescent cells (blue) in BRL cells treated with TGF-β1 (20 ng/mL) for 4 days to establish a senescence model. **(F)** qPCR analysis of *Cdkn1a* and *Cdkn2a* mRNA levels in BRL cells after TGF-β1 treatment. **(G)** Western blot analysis of P16 and P21 protein expression in BRL cells after TGF-β1 treatment. **(H)** qPCR analysis of relative mRNA levels of SASP factors (*Il-1a*, *Il-1b*, *Il-6*, *Ifn-γ*, *Tnf-α*) in BRL cells after TGF-β1 treatment. **(I)** qPCR analysis of *Cdkn2a* and *Cdkn1a* mRNA levels in TMBIM1-knockdown BRL cells after TGF-β1 treatment. **(J)** Western blot analysis of P16 and P21 protein expression in TMBIM1-knockdown BRL cells after TGF-β1 treatment. **(K)** SA-β-gal staining detecting senescent cells (blue) in BRL cells treated with TGF-β1 for 4 days. All data are presented as mean ± SD (n=3). *p < 0.05, **p < 0.01, ***p < 0.001, ****p < 0.0001.

**Supplementary Figure 4.** **(A)** Expression levels of TMBIM1-positive cells at various stages of DEN-induced carcinogenesis. **(B)** Expression levels of TMBIM1-positive cells following AVV8-TMBIM1 injection. **(C)** Quantity of senescent cells at various stages of DEN-induced carcinogenesis. **(D, E)** Expression levels of CD133 and Sox-9 positive cells during the carcinogenic phases at 0 weeks and 12 weeks. **(F)** The number of senescent cells at 12 weeks post-cancer induction. **(G, H, I)** The expression levels of senescence-associated markers P21, P16, and the proportion of P21/γ-H2AX-positive cells at 12 weeks post-cancer induction. **(J)** The number of senescent cells in primary cell cultures at 12 weeks following cancer induction. **(K, L)** The expression of CD133 and Sox-9 positive cells at 12 weeks post-cancer induction. **(M)** The area exhibiting fibrosis-associated α-SMA positivity at 12 weeks post-cancer induction. **(N)** The expression of inflammation-associated CD68 cells at 12 weeks following cancer induction. **(O, P)** The expression of apoptosis-associated markers Caspase 8 and Fas/CD95 at 12 weeks post-cancer induction. **(Q)** Quantification of senescent cells in TMBIM1-overexpressing cells treated with LPS. All data are expressed as mean±SD. n=3. **p<0.01, ***p<0.001 and ****p<0.0001.

**Supplementary Figure 5. Rab9a knockdown reverses the antitumor effects of TMBIM1. (A)** Western blot analysis of Rab9a protein expression in mouse liver tissues. **(B)** Representative macroscopic images of livers from Rab9a-knockdown, Rab9a-knockdown plus TMBIM1-overexpression, and TMBIM1-overexpression groups. **(C)** HE staining of liver tissues from different groups to assess pathological changes. **(D)** Body weights of mice in different experimental groups. **(E)** Quantitative analysis of tumor sizes in liver tissues from different groups. **(F)** Schematic of the subcutaneous tumor formation model established by co-injection of senescent cells and tumor cells. Subcutaneous tumors from different groups are shown after dissection. **(G)** Quantitative analysis of subcutaneous tumor weights in different experimental groups. All data are presented as mean ± SD (n=3). *p < 0.05, **p < 0.01, ***p < 0.001, ****p < 0.0001, ns: not significant.

**Table S1. Real-time PCR primers**

| Rat gene |  | Sequence 5'--3' |
| --- | --- | --- |
| ***Tmbim1*** | F | AGTGAGGAGGAGAGAGCAGG |
|  | R | TGGCAGCAGACAAGGATCAG |
| ***Cdkn2a*** | F | AGTGAGGAGGAGAGAGCAGG |
|  | R | TGGCAGCAGACAAGGATCAG |
| ***Cdkn1a*** | F | TGTTCCACACAGGAGCAAAG |
|  | R | AACACGCTCCCAGACGTAGT |
| ***Il-1α*** | F | GCTTGAGTCGGCAAAGAAAT |
|  | R | GACAGATGGTCAATGGCAGA |
| ***Il-1β*** | F | CACCTCTCAAGCAGAGCACAG |
|  | R | GGGTTCCATGGTGAAGTCAAC |
| ***Il-6*** | F | TCCTACCCCAACTTCCAATGCTC |
|  | R | TTGGATGGTCTTGGTCCTTAGCC |
| ***Tnf-α*** | F | CAAATGGGCTCCCTCTCATC |
|  | R | TTGGTGGTTTGCTACGACG |
| ***Ifn-γ*** | F | CGGCACAGTCATTGAAAGCCTA |
|  | R | GTTGCTGATGGCCTGATTGTC |
| ***Gapdh*** | F | CAACTCCCTCAAGATTGTCAGCAA |
|  | R | GGCATGGACTGTGGTCATGA |
| ***Tgfbr1*** | F | ACTCCCAACTACAGAAAAGCA |
|  | R | GGTGAATGACAGTGCGGTTA |
| ***Tgfbr2*** | F | CCAAGTCGGTTAACAGCGAT |
|  | R | GTCGTTCTTCCTCCACACG |

**Table S2. Primers for plasmid construction.**

**S2.1 Rat_*Tmbim1* shRNA oligo sequences**

| Oligo name | Oligomeric single-stranded DNA sequences 5' to 3' |
| --- | --- |
| **Primer-NC-T** | GATCTGTTCTCCGAACGTGTCACGTTTCAAGAGAACGTGACACGTTCGGAGAATTTTTTC |
| **Primer-NC-B** | AATTGAAAAAATTCTCCGAACGTGTCACGTTCTCTTGAAACGTGACACGTTCGGAGAACA |
| **Primer-T1** | gatccGTGACCGGAAAGTCCGACATACCTCGAGGTATGTCGGACTTTCCGGTCATTTTTT |
| **Primer-B1** | aattAAAAAATGACCGGAAAGTCCGACATACCTCGAGGTATGTCGGACTTTCCGGTCACg |
| **Primer-T2** | gatccGTGTCCTATGCTGTCTTCATTGCTCGAGCAATGAAGACAGCATAGGACATTTTTT |
| **Primer-B2** | aattAAAAAATGTCCTATGCTGTCTTCATTGCTCGAGCAATGAAGACAGCATAGGACACg |
| **Primer-T3** | gatccACAGGCGCTATTTCCAGTATGCTCGAGCATACTGGAAATAGCGCCTGTTTTTTT |
| **Primer-B3** | aattAAAAAAACAGGCGCTATTTCCAGTATGCTCGAGCATACTGGAAATAGCGCCTGTg |

**S2.2 Rat_*Tmbim1*-3×Flag oligo sequences**

| Oligo name | Oligomeric single-stranded DNA sequences 5' to 3' |
| --- | --- |
| **81376FW-108367** | CGAATTCGAAGTATACCTCGAGGCCACCATGTCCAATCCCA |
| **81376RW-108368** | CATGGTCTTTGTAGTCGGATCCGTCTCGATTTCCTACAAGCTGGAGC |

**S2.3 Rat_*Rab9a* -HA oligo sequences**

| oligo name | Oligomeric single-stranded DNA sequences 5' to 3' |
| --- | --- |
| **84124FW-108369** | CTACCGGACTCAGATCTCGAGGCCACCATGGCAGGAAAATC |
| **84124RW-108370** | GTTATCTAGATCCGGTGGATCCTCAAGCGTAATCTGGAACATCG |

**S2.4 Rat_*Tmbim1*-eGFP-3×Flag oligo sequences**

| oligo name | Oligomeric single-stranded DNA sequences 5' to 3' |
| --- | --- |
| **84123FW-98979** | GCTACCGGACTCAGATCTCGAGGCCACCATGTCCAATCCCA |
| **84123RW-98980** | CACCATACCACTACCGAATTCGTCTCGATTTCCTACAAGCTGGA |

**S2.5 Rat_*Rab9a* shRNA oligo sequences**

| Oligo name | Oligomeric single-stranded DNA sequences 5' to 3' |
| --- | --- |
| **Primer-NC-T** | GATCTGTTCTCCGAACGTGTCACGTTTCAAGAGAACGTGACACGTTCGGAGAATTTTTTC |
| **Primer-NC-B** | AATTGAAAAAATTCTCCGAACGTGTCACGTTCTCTTGAAACGTGACACGTTCGGAGAACA |
| **Primer-T1** | gatccAGTTGGCAAGAGTTCTCTTATCTCGAGATAAGAGAACTCTTGCCAACTTTTTTT |
| **Primer-B1** | aattAAAAAAAGTTGGCAAGAGTTCTCTTATCTCGAGATAAGAGAACTCTTGCCAACTg |
| **Primer-T2** | gatccGTCCCAGCTCTTCCACACAATACTCGAGTATTGTGTGGAAGAGCTGGGATTTTTT |
| **Primer-B2** | aattAAAAAATCCCAGCTCTTCCACACAATACTCGAGTATTGTGTGGAAGAGCTGGGACg |
| **Primer-T3** | gatccGAACAGATATGTAACCAATAACTCGAGTTATTGGTTACATATCTGTTCTTTTTT |
| **Primer-B3** | aattAAAAAAGAACAGATATGTAACCAATAACTCGAGTTATTGGTTACATATCTGTTCg |

**S2.6 *Moses*_*Rab9a* shRNA oligo sequences**

| Oligo name | Oligomeric single-stranded DNA sequences 5' to 3' |
| --- | --- |
| **Primer-T1** | gatccAGTTGGCAAGAGTTCTCTTATCTCGAGATAAGAGAACTCTTGCCAACT TTTTTT |
| **Primer-B1** | aattAAAAAAAGTTGGCAAGAGTTCTCTTATCTCGAGATAAGAGAACTCTTGCCAACTg |
| **Primer-T2** | gatccGAACAGATATGTAACCAATAACTCGAGTTATTGGTTACATATCTGTTCTTTTTT |
| **Primer-B2** | aattAAAAAAGAACAGATATGTAACCAATAACTCGAGTTATTGGTTACATATCTGTTCg |
| **Primer-T3** | gatccGTCTCAGCTCTTCCACACAATACTCGAGTATTGTGTGGAAGAGCTGAGATTTTTT |
| **Primer-B3** | aattAAAAAATCTCAGCTCTTCCACACAATACTCHAGTATTGTGTGGAAGAGCTGAGACg |

**Table S3. Demographic and baseline characteristics of 70 HCC patients.**

| **Factors** | **Value** | **Percent** |
| --- | --- | --- |
| **Age** | **50.5** **± 10.2** |  |
| **Gender (****male/female)** | **55/15** | **78.6/21.5** |
| **Maximum tumor diameter** | **5.21 ± 2.58** |  |
| **Tumor number (multiple)** | **11** | **15.7** |
| **Tumor thrombosis** | **9** | **12.9** |
| **Alpha fetoprotein (ng/mL)** | **300 ± 411** |  |
