# Supplementary figures and images for: Transmembrane BAX inhibitor motif containing 1 inhibition of lysosomal degradation of TGF-β receptor 1 suppresses cellular senescence and hepatocarcinogenesis
Source: J Biol Chem. 2025 Nov 4;301(12):110904. doi: 10.1016/j.jbc.2025.110904 (PMC12702014; doi:10.1016/j.jbc.2025.110904)

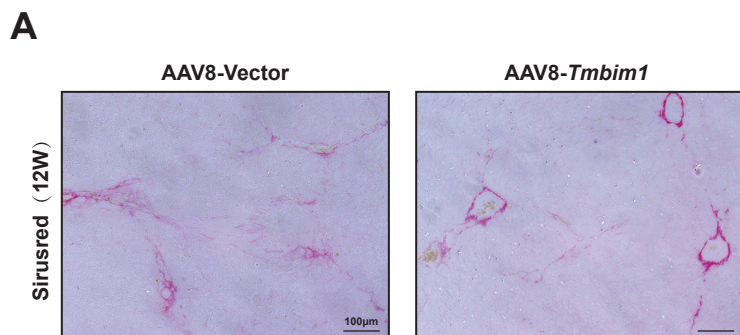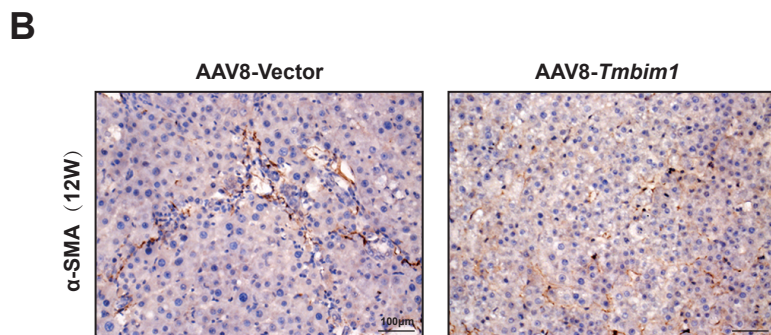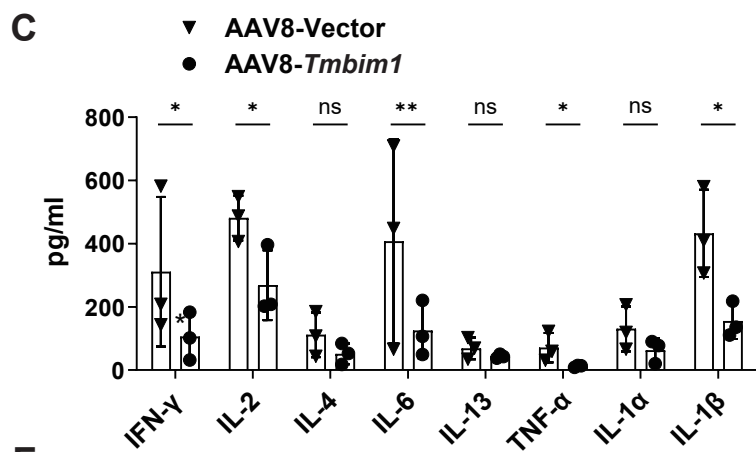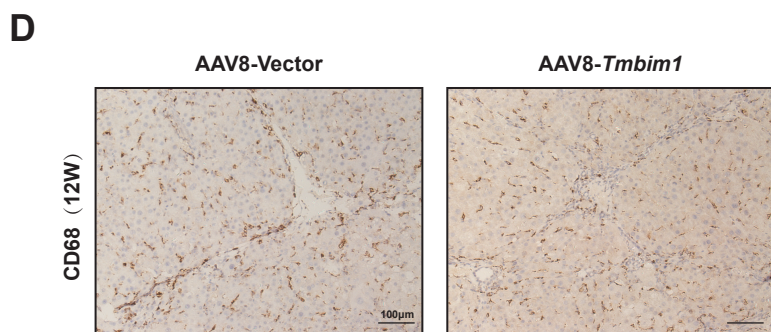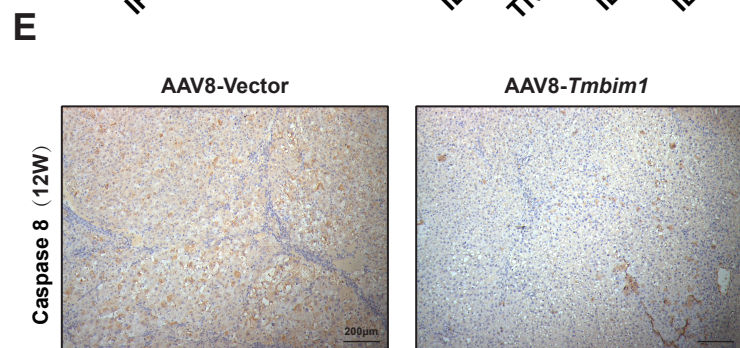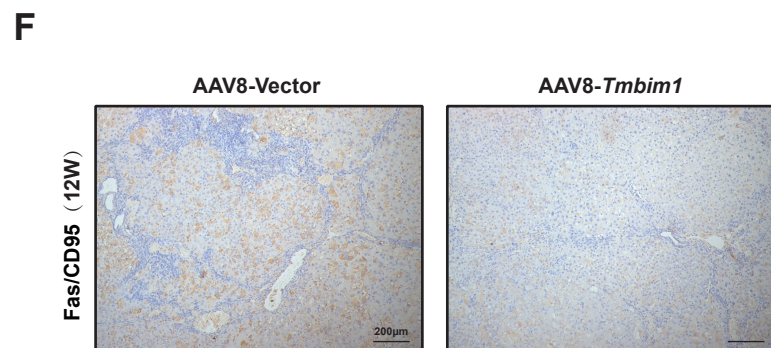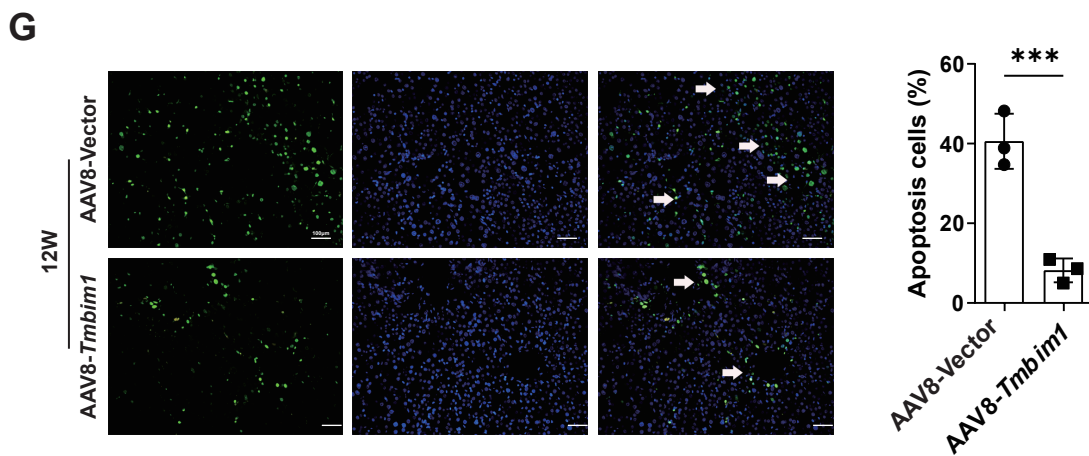

Supplement: Supplementary Figure 1 [file mmc2.pdf]

**A**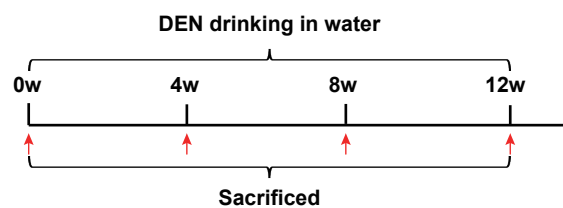**B**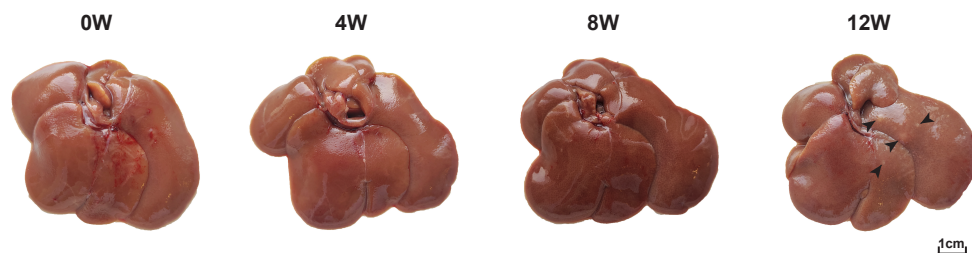**C**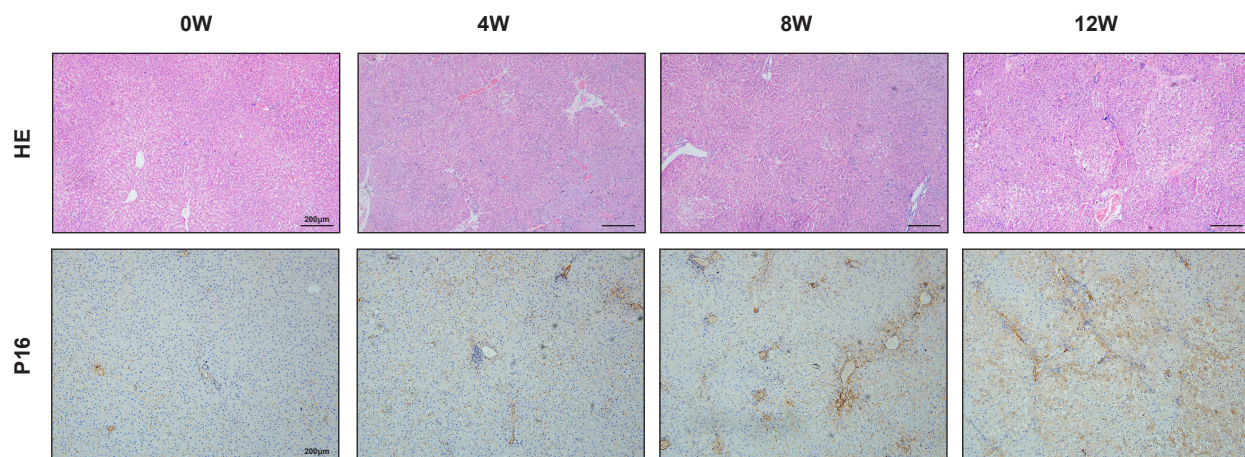**D**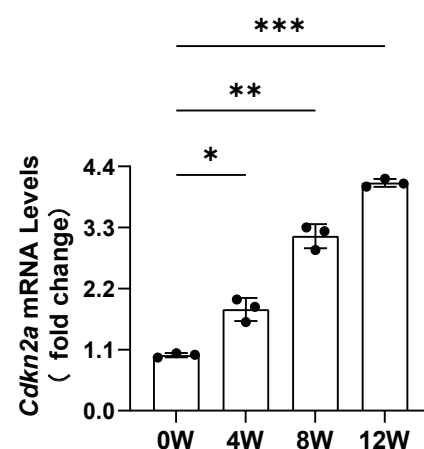**E**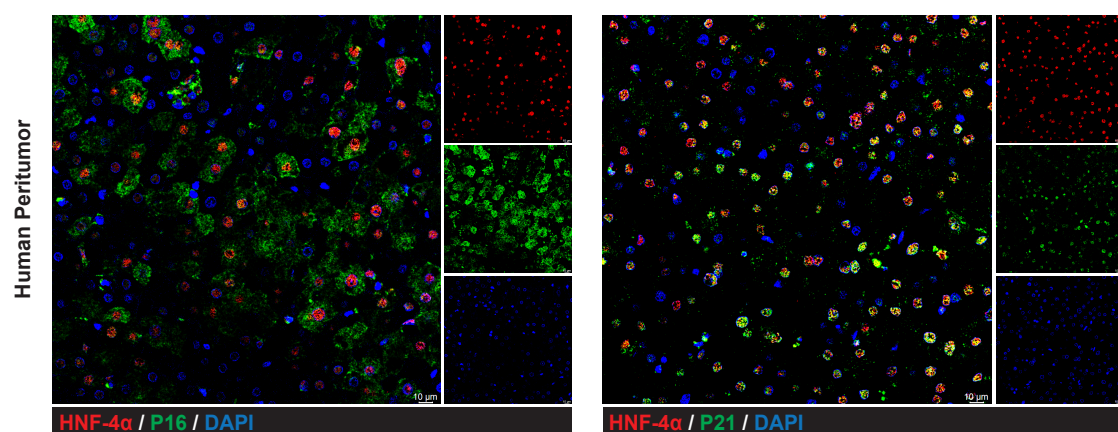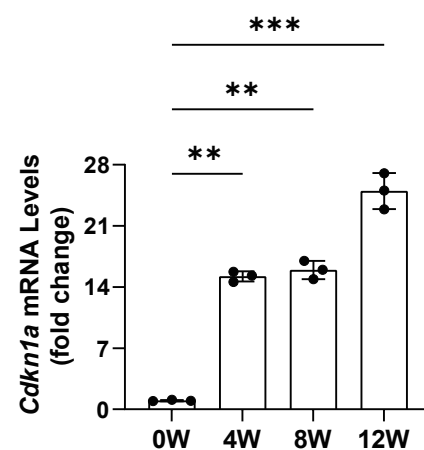**F**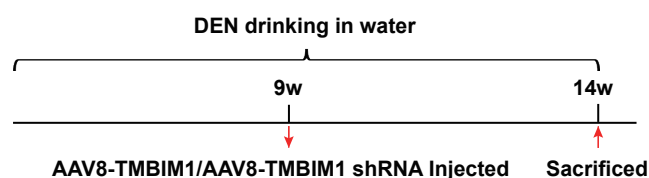**H**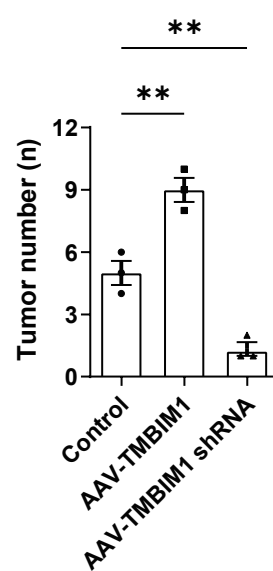**I**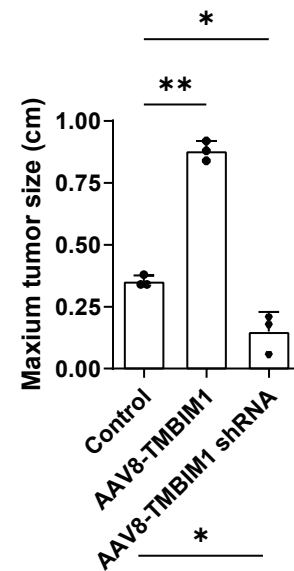**G**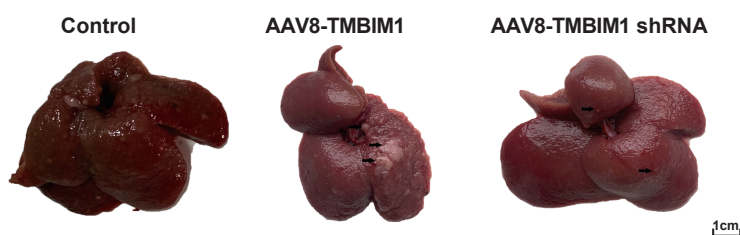**J**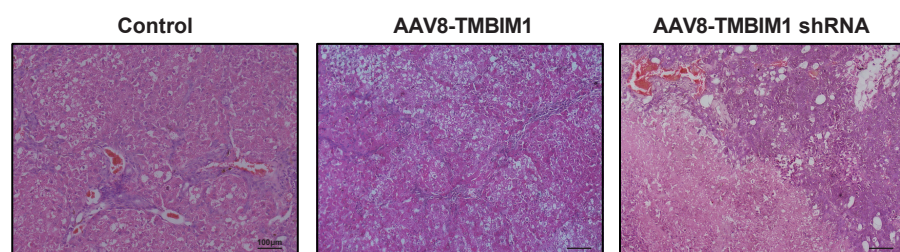**K**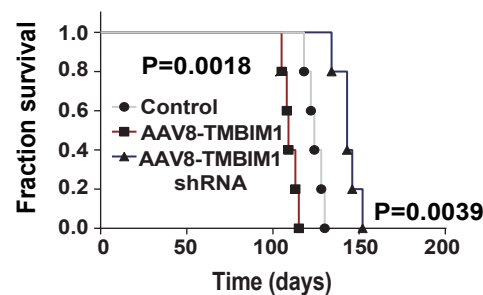**L**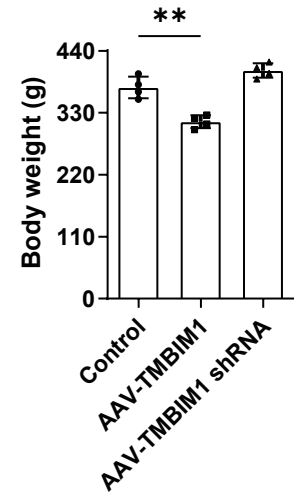

Supplement: Supplementary Figure 2 [file mmc3.pdf]

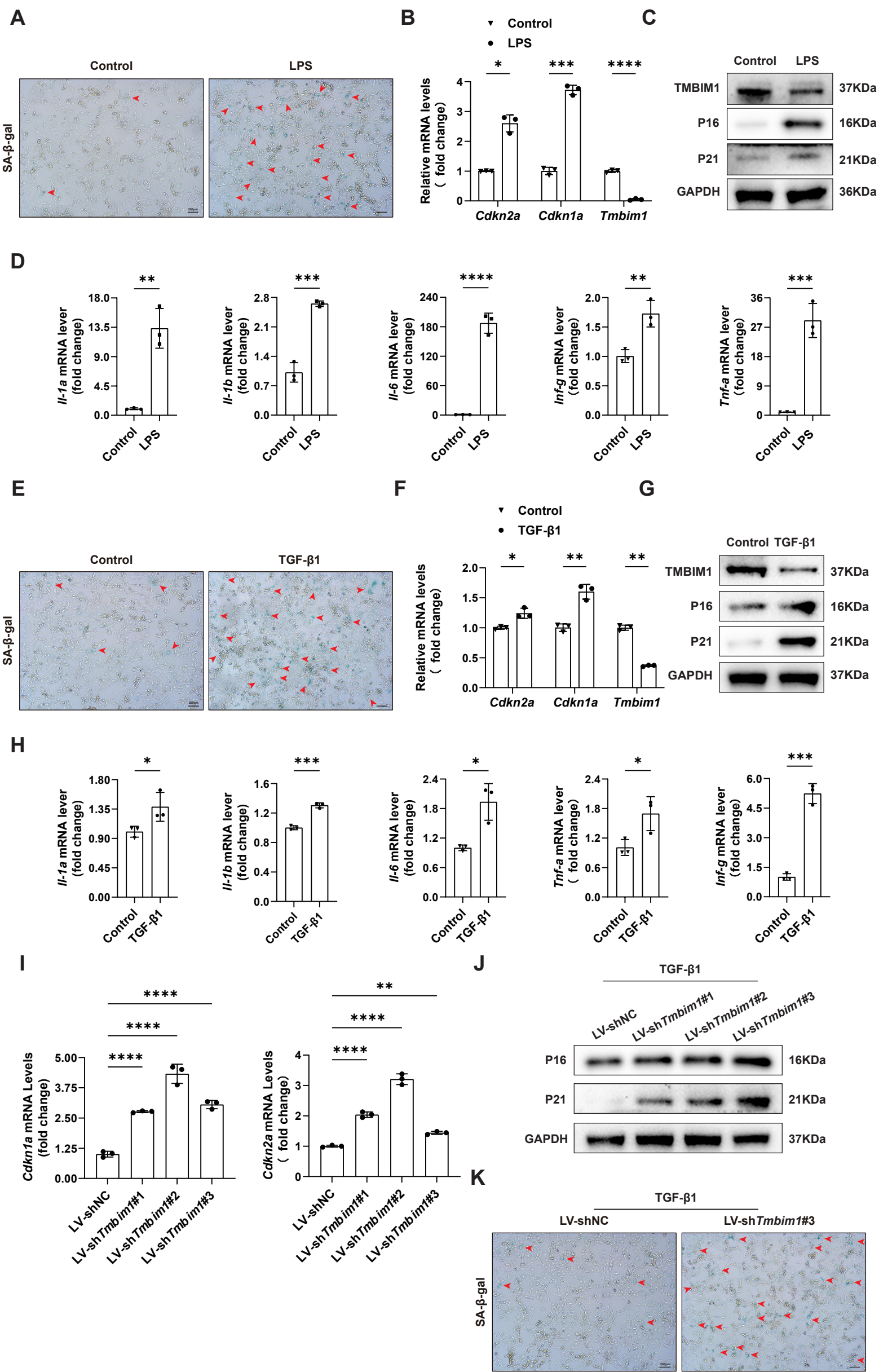

Supplement: Supplementary Figure 3 [file mmc4.pdf]

**A**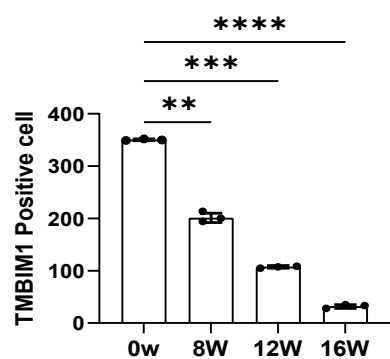**B**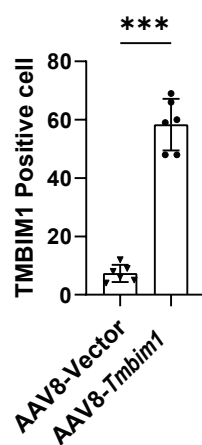**C**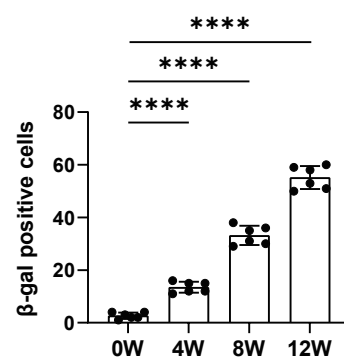**D**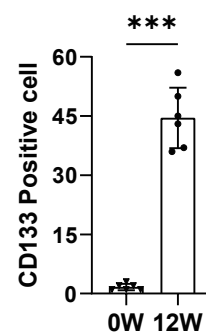**E**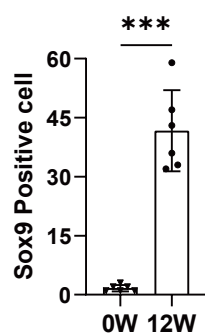**F**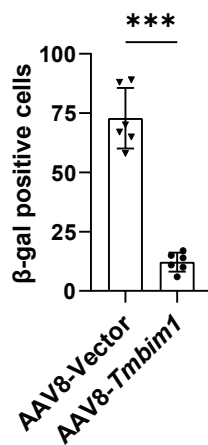**G**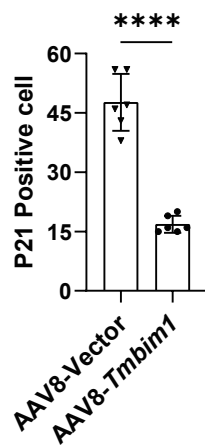**H**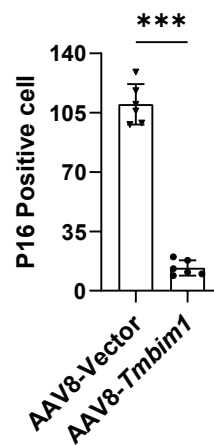**I**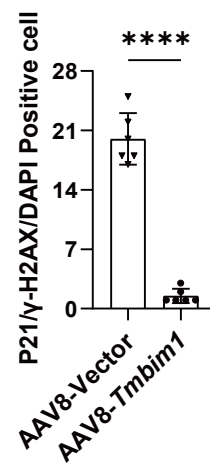**J**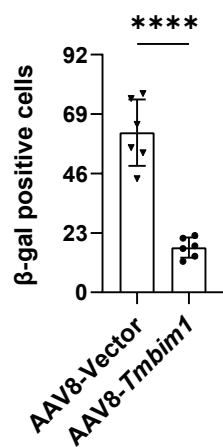**K**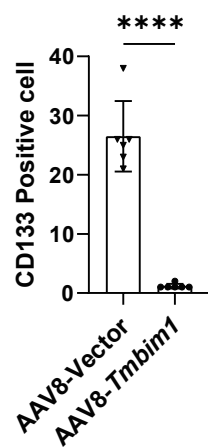**L**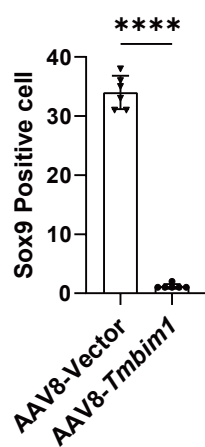**M**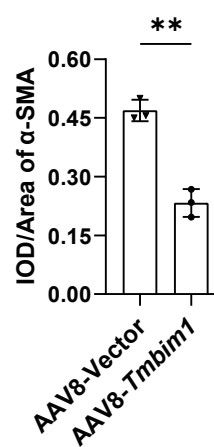**N**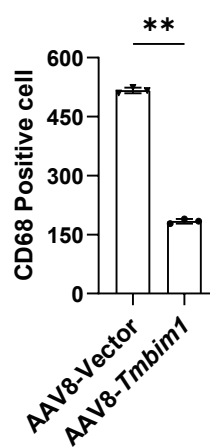**O**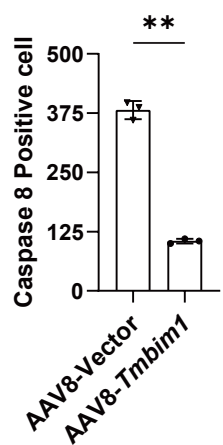**P**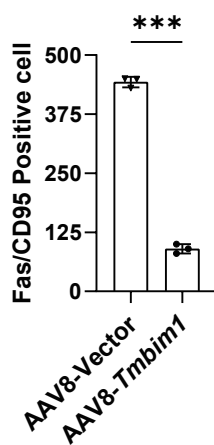**Q**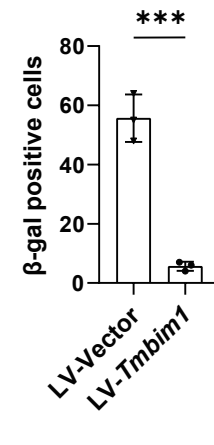

Supplement: Supplementary Figure 4 [file mmc5.pdf]
