# Supplementary material for: Transmembrane BAX inhibitor motif containing 1 inhibition of lysosomal degradation of TGF-β receptor 1 suppresses cellular senescence and hepatocarcinogenesis
Source: J Biol Chem. 2025 Nov 4;301(12):110904. doi: 10.1016/j.jbc.2025.110904 (PMC12702014; doi:10.1016/j.jbc.2025.110904)
Supplement: Supplementary Figure 5 [file mmc6.pdf]

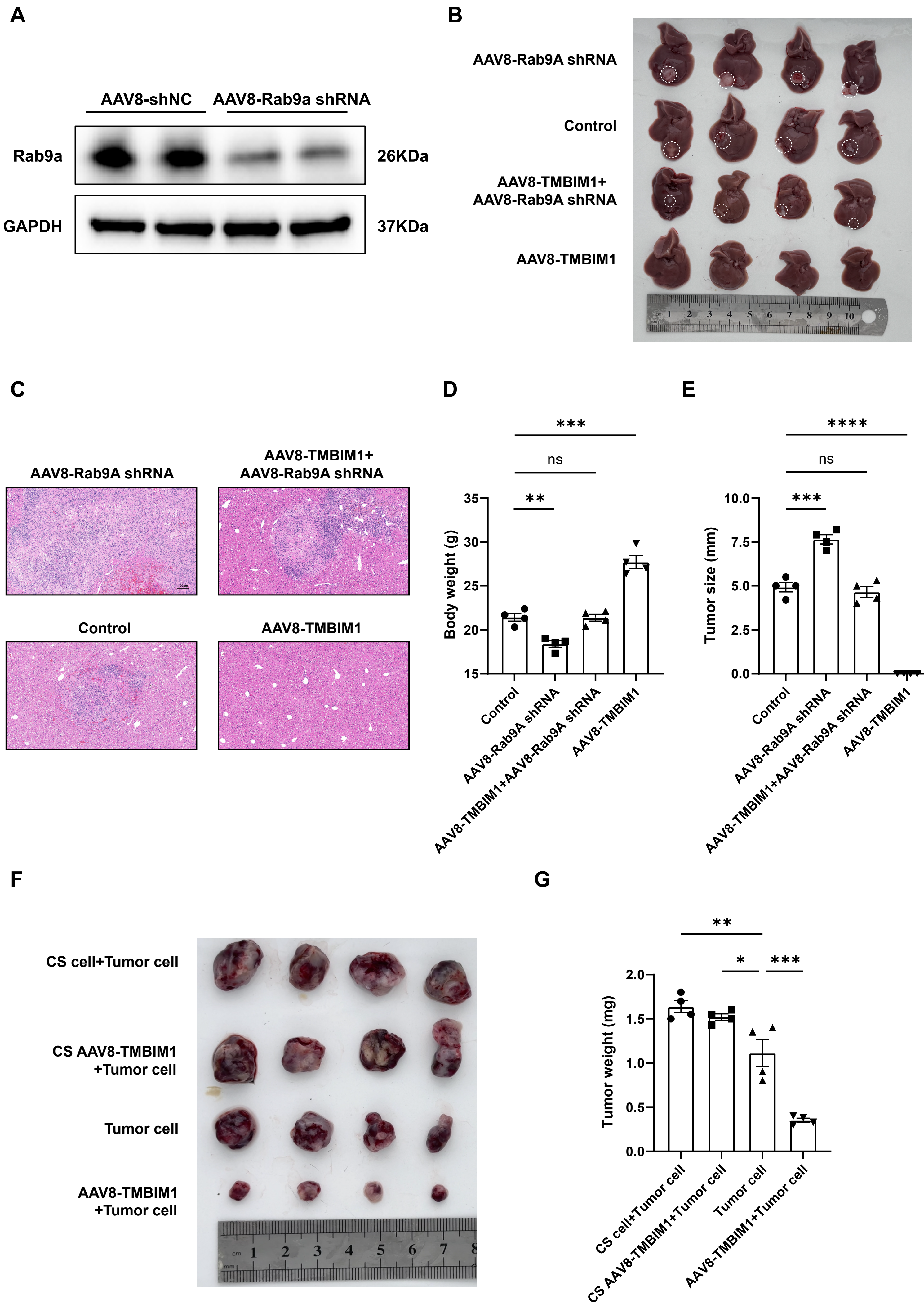
A
AAV8-shNC
AAV8-Rab9a shRNA
Rab9a
26KDa
GAPDH
37KDa
B
AAV8-Rab9A shRNA
Control
AAV8-TMBIM1+
AAV8-Rab9A shRNA
AAV8-TMBIM1
C
AAV8-Rab9A shRNA
AAV8-TMBIM1+
AAV8-Rab9A shRNA
Control
AAV8-TMBIM1
D
***
ns
**
Body weight (g)
35
30
25
20
15
Control
AAV8-Rab9A shRNA
AAV8-TMBIM1+AAV8-Rab9A shRNA
AAV8-TMBIM1
E
****
ns
***
Tumor size (mm)
10.0
7.5
5.0
2.5
0.0
Control
AAV8-Rab9A shRNA
AAV8-TMBIM1+AAV8-Rab9A shRNA
AAV8-TMBIM1
F
CS cell+Tumor cell
CS AAV8-TMBIM1
+Tumor cell
Tumor cell
AAV8-TMBIM1
+Tumor cell
G
**
*
***
Tumor weight (mg)
2.0
1.5
1.0
0.5
0.0
CS cell+Tumor cell
CS AAV8-TMBIM1+Tumor cell
Tumor cell
AAV8-TMBIM1+Tumor cell
